# Safety, tolerability, pharmacokinetics and pharmacodynamics of single oral doses of BI 187004, an inhibitor of 11beta-hydroxysteroid dehydrogenase-1, in healthy male volunteers with overweight or obesity

**DOI:** 10.1186/s40842-021-00130-x

**Published:** 2021-08-15

**Authors:** Susanna Bianzano, Tim Heise, Arvid Jungnik, Cornelia Schepers, Corinna Schölch, Ulrike Gräfe-Mody

**Affiliations:** 1 Boehringer Ingelheim International GmbH, Binger Strasse 173, 55216 Ingelheim am Rhein, Germany; 2 Profil, Neuss, Germany; 3 Boehringer Ingelheim Pharma GmbH & Co. KG, Biberach, Germany; 4 Boehringer Ingelheim Pharma GmbH & Co. KG, Ingelheim, Germany

**Keywords:** 11beta-Hydroxysteroid dehydrogynase-1 inhibitor, Single rising dose, Pharmacokinetics, Pharmacodynamics, Type 2 diabetes, BI 187004

## Abstract

**Background:**

The study characterizes safety, tolerability, pharmacokinetic and pharmacodynamic profiles of single rising doses of the 11beta-hydroxysteroid dehydrogenase-1 (11beta-HSD1) inhibitor BI 187004 in healthy men with overweight or obesity.

**Methods:**

This was a randomized, double-blind, parallel group, placebo-controlled study with administration of 2.5–360 mg BI 187004 or placebo once daily as single dose in 72 healthy male volunteers with overweight or obesity.

Assessments included 11beta-HSD1 inhibition in the liver (assessed indirectly by urinary tetrahydrocortisol/tetrahydrocortisone ratio) and in subcutaneous adipose tissue ex vivo and determination of hypothalamus–pituitary–adrenal axis hormones.

**Results:**

BI 187004 was well tolerated and safe in all tested dose groups. The incidence of drug-related adverse events was 16.7% (n = 9) for all 9 BI 187004 dose groups and 5.9% (n = 1) for placebo. All treatment groups were similar concerning kind and intensity of adverse events. No clinically relevant deviations in clinical laboratory or ECG parameters were reported. Exposure of BI 187004 increased non-proportionally over the entire dose range tested. The geometric mean apparent terminal half-life decreased from 33.5 h (5 mg) to 14.5 h (160 mg) remaining stable up to 360 mg. Renal excretion of BI 187004 was low (3–5%). Urinary tetrahydrocortisol/tetrahydrocortisone ratio decreased, indicating liver 11beta-HSD1 inhibition. Median inhibition of 11beta-HSD1 in subcutaneous adipose tissue biopsies following single dosing ranged from 86.8% (10 mg) to 99.5% (360 mg) after 10 h and from 59.4% (10 mg) to 98.6% (360 mg) after 24 h.

**Conclusions:**

BI 187004 as single dose was safe and well tolerated and is suitable for once daily dosing. There was significant, sustained 11beta-HSD1 inhibition in liver and adipose tissue.

**Trial registration:**

ClinicalTrials.gov, NCT01587417, registered on 26-Apr-2012.

**Supplementary Information:**

The online version contains supplementary material available at 10.1186/s40842-021-00130-x.

## Introduction

Activation of the glucocorticoid receptor with overproduction of active cortisol is thought to result in multiple clinical characteristics of the metabolic syndrome including insulin resistant type 2 diabetes mellitus (T2DM), visceral obesity, dyslipidemia and arterial hypertension [1]. Besides an increase in plasma cortisol levels also tissue-specific changes in cortisol metabolism might promote development of the metabolic syndrome [2]. The enzyme 11beta-hydroxysteroid dehydrogenase-1 (11beta-HSD1) is highly expressed in liver and adipose tissue (AT) and is responsible for the conversion of the biologically inactive cortisone to active cortisol [3]. Currently, it is thought that the main factor to regulate 11beta-HSD1 activity in liver and AT could be body weight. However, there are conflicting data on the interaction of 11beta-HSD1 expression, obesity and T2DM: In obese subjects with and without T2DM 11beta-HSD1 was found to be upregulated in subcutaneous AT when compared to healthy lean controls [4–6]. In obese men with T2DM 11beta-HSD1 activity in AT was increased whereas 11beta-HSD1 activity in liver was sustained [7, 8]. In contrast, euglycemic obese subjects showed decreased 11beta-HSD1 activity in liver but increased enzyme activity in subcutaneous AT [9]. As summarized in [10] in patients with T2DM and obesity the acute effect of 11beta-HSD1 inhibition is most likely improvement of glucose metabolism by reduction of glucocorticoid-mediated hepatic glucose output. Inhibition of 11beta-HSD1 in AT is thought to result in long-term effects with reduction of body weight and improvement of dyslipidemia and arterial hypertension. Thus, 11beta-HSD1 inhibition should be targeted in both, liver as well as AT and focus on a target population with T2DM and obesity.

Proof of concept for the 11beta-HSD1 inhibitor class as an anti-diabetic modality was demonstrated with INCB13739 treatment over 12 weeks in subjects with T2DM, who were failing metformin monotherapy [11]. However, despite efficacious HbA1c lowering and a good safety profile development of this compound was for unknown reasons not further pursued. Since then, most 11beta-HSD1 inhibitor development programs have been discontinued, predominantly due to insufficient efficacy in terms of glucose lowering. Nevertheless, the tissue-specific improvement of cortisol metabolism via 11beta-HSD1 inhibition is still of high interest in treatment of T2DM and parameters the metabolic syndrome, mainly obesity and hyperlipidemia. It seems to be crucial to have a compound which is safe and able to inhibit 11beta-HSD1 to a high extent in liver and AT and BI 187004 is expected to fulfill these prerequisites.

The purpose of this study was to evaluate the safety, tolerability, pharmacokinetics (PK) and pharmacodynamics (PD) of single rising doses of the novel 11beta-HSD1 inhibitor BI 187004 up to 360 mg once daily in healthy male volunteers with overweight or obesity.

## Materials and methods

### Participants

Participants were male healthy volunteers with overweight or obesity aged 18–55 years with a body mass index (BMI) ≥ 28 kg/m^2^ for participants which received subcutaneous AT biopsies (10–360 mg group), BMI ≥ 25 kg/m^2^ for participants which did not receive AT biopsies (2.5–5 mg group). To reduce a safety risk for a molecule which has never been tested in humans in this first trial women were not included. The main exclusion criteria were any evidence of a clinically relevant concomitant disease, and gastrointestinal, hepatic, renal, respiratory, cardiovascular, metabolic, immunological or hormonal disorders besides hyperlipidemia. To exclude impaired fasting glucose and diabetes prior to the study the following exclusion criteria were defined: (i) any evidence of a clinically relevant concomitant disease, (ii) any laboratory value outside the reference range that is of clinical relevance, (iii) Fasted plasma glucose ≥ 126 mg/dL (7.0 mmol/l) in repeated tests during screening.

The study was undertaken according to the Declaration of Helsinki and Good Clinical Practice principles. The protocol was approved by local independent ethics committee (Ärztekammer Nordrhein, Düsseldorf, Germany). All participants provided written informed consent before participation. The clinical trial registry number is NCT01587417 (www.clinicaltrials.gov).

### Study design and procedures

This was a randomized, double-blind, placebo-controlled, single rising dose study conducted at a single center in Germany. A total of 72 healthy male volunteers entered and 71 completed the study. Subjects were treated in 9 sequentially ascending dose groups of 2.5, 5, 10, 20, 40, 80, 160, 240, and 360 mg of BI 187004 or matching placebo. Within each dose group, 6 subjects were treated with a single dose of BI 187004, and 2 subjects received placebo (except for the 5 mg dose group, where only 1 subject received placebo). The subjects were admitted to the study center on day -2, 36 h prior to the treatment and were discharged on day 2. Follow-up on an ambulatory basis was performed between 11 and 15 days after study drug administration. The active drug was provided as a powder for oral solution (PFOS). At the time of use the oral solution for dosing was prepared using the PFOS and a co-supplied aqueous solvent containing HP-beta-cyclodextrin 100 mg/mL. The lowest dose level solutions were prepared by further dilution of the lower concentration solution with additional solvent. Subcutaneous AT sample collection occurred at three different time points for each subject on day -1 (baseline), day 1 (10 h after single dose) and day 2 (24 h after single dose) via incision biopsy in the umbilical region.

Plasma samples for PK analyses were obtained from day 1 to day 2 (pre-dose, 0.5, 1, 1.5, 2, 2.5, 3, 4, 5, 6, 8, 10, 12, 24, 36 h), day 3 to day 5, day 11 to day 15 (in the morning). 24-h urinary collection for PK and PD analysis was obtained from day -1 to day 4 in 4-h to 12-h intervals (relative to drug administration).

BI 187004 concentrations in plasma and urine samples were measured with validated bioanalytical methods using a high-performance liquid chromatography-tandem mass spectrometry (LC–MS/MS) method. The assay method consists of a solid supported liquid–liquid extraction of human plasma, coupled with quantitative LC–MS/MS determination of the extracted samples and was validated for the concentration range of BI 187004 of 3—3,000 nmol/L. The BI 187004 plasma concentration data were generated from 11 analytical runs. For each accepted run, the acceptance criteria for the calibration standards and quality controls were met.

The BI 187004 concentrations in urinary samples were measured using a LC–MS/MS method which was validated for the concentration range of BI 187004 of 5—5,000 nmol/L. The BI 187004 urine concentration data were generated from 5 analytical runs. For each accepted run, the acceptance criteria for the calibration standards and quality controls were met.

The acceptance criteria for the plasma and urine assay were (i) a regression coefficient (R^2^) of ≥ 0.98; (ii) at least 75% of calibration standards with ≤ 15%RE (relative error / accuracy) and (iii) each accepted curve contained at least 6 concentration levels.

### Safety assessments

Safety and tolerability of BI 187004 were assessed in a descriptive way using the following investigations: recording of adverse events, clinical laboratory parameters (haematology, coagulation, enzymes, substrates, electrolytes, hormones, and urinalysis), vital signs (blood pressure, puls rate), 12-lead ECG (electrocardiogram) with special attention to QTc prolongation, and physical examination (occurrence of findings).

### Measurement of urinary corticosteroids

The urinary samples for determination of urinary free cortisol (UFF), urinary free cortisone (UFE), urinary total cortisol (UTF), urinary total cortisone (UTE), and their metabolites 5alpha- or allo-tetrahydrocortisol (aTHF), 5beta-tetrahydrocortisol (THF), and tetrahydrocortisone (THE) were analyzed using a validated LC–MS/MS method.

### Concentration measurement of BI 187004 in adipose tissue

The BI 187004 concentrations in human AT samples were measured using a protein-precipitation extraction of homogenized human AT, coupled with quantitative LC–MS/MS determination of the extracted samples. The assay was qualified for the BI 187004 concentration range of 2.5—10,000 ng/g. The BI 187004 AT concentration data for study samples were generated from seven analytical runs. For each run, the acceptance criteria for the calibration standards and QC samples were met. Acceptance criteria for all sample analysis runs were ≤ 25% RSD (relative standard deviation / precision) and ± 25% RE (relative error / accuracy) for calibration standards and quality control samples.

### *Ex vivo* measurement of 11beta-HSD1 activity

Inhibition of 11beta-HSD1 in AT was measured by conversion of deuterized (d2)-cortisone to d2-cortisol ex vivo in subcutaneous AT biopsies. Immediately after taking the biopsies with an open incision, tissue samples were cut into fragments, placed into 48-well tissue culture plates and incubated in assay buffer. A triplicate set of tissue fragments was incubated in media with d2-cortisone and 1 μM test substance to establish the background level of d2-cortisol. After overnight incubation, about 80 μL of the supernatant were snap frozen and stored at ≤ -60 °C. Then, d2-cortisone and d2-cortisol were measured using a validated LC–MS/MS method with the formation of d2-cortisol being an indirect indicator for enzyme activity.

### Statistical analysis

11beta-HSD1 inhibition in AT was calculated as individual percent change from baseline to day 1 and day 2 of the absolute deuterized cortisol measurements and expressed in medians. Safety laboratory parameters including hormone measurements are given as mean ± standard deviation, PK parameters as geometric mean (gMean) ± geometric coefficient of variation (%gCV), except for t_max_ (time of maximum observed drug concentration), which is given as median ± range. Methods for the determination of PK parameters were described previously [12]. Dose proportionality of BI 187004 was explored using a power model. The placebo corrected percentage change from baseline of the data shown in Supplementary Table 1 and 2 was analyzed statistically by an ANCOVA model with baseline as covariate on the logarithmic scale. Placebo correction was achieved by estimating mean differences between each dose of BI 187004 and placebo with the corresponding 90% confidence interval (CI). The results were back-transformed to the original scale resembling post dose/baseline ratios for each dose of BI 187004 model.

## Results

### Subject disposition and demographics

A total of 71 male subjects were treated in 9 sequential dose groups with one single dose of BI 187004 in ascending order of 2.5, 5, 10, 20, 40, 80, 160, 240, and 360 mg and placebo. All treated subjects completed the trial according to the clinical trial protocol. All subjects were white men with a mean (± SD) age of 40.8 ± 8.8 years. Body weight (mean ± SD) was 99.61 ± 13.29 kg and body mass index (BMI, mean ± SD) was 30.17 ± 2.73 kg/m^2^. There were no relevant differences seen between dose groups with respect to demographic characteristics. No relevant medical conditions and concomitant therapies were reported. Baseline metabolic characteristics are summarized in Supplementary table 3.

### Safety

In total 17 out of the 71 subjects (23.9%) reported at least 1 adverse event (AE) during the trial, with 15 subjects (27.8%) reporting events following BI 187004 treatment and 2 subjects (11.8%) reporting AEs following placebo. AEs that were considered drug-related by the investigator occurred in 9 subjects (16.7%) in total for all BI 187004 dose groups and in 1 subject (5.9%) for placebo. The treatment groups were similar with regard to the kind and intensity of AEs. All subjects recovered from their AEs by the end of the trial. No deaths, no AEs leading to discontinuation, and no serious AEs occurred during the trial. The AE with the highest incidence was headache, observed in 3 out of 54 subjects (5.6%) receiving BI 187004 and in 1 out of 17 subjects (5.9%) receiving placebo. All other AEs occurred with a frequency of less than 5%. There were no notable findings with respect to clinical laboratory evaluations, vital signs, and ECG recordings. No hypoglycemic episodes were reported.

As summarized by Stomby et al. [13] following 11beta-HSD1 inhibition serum cortisol levels decrease with a compensatory increase of adrenocorticotropic hormone (ACTH) leading to increased activity of the hypothalamus–pituitary–adrenal (HPA) axis. This is followed by increased adrenal activity leading to stable cortisol levels and increased androgens. At all BI 187004 dose levels, cortisol levels decreased and dehydroepiandrosterone-sulphate (DHEA-s) slightly increased in a dose-independent manner (Table 1). ACTH and androstenedione levels increased in dose groups ≥ 20 mg compared with placebo and slightly decreased or remained unchanged thereafter (Table 1). The observed differences from baseline in total testosterone and sex-hormone binding globulin (SHBG) levels were still within the respective reference ranges (Table 1). In addition, total urinary corticosteroid excretion increased ranging from 130.3% (2.5 mg) to 197.5% (80 mg) across all BI 187004 dose levels suggesting an effect of BI 187004 on the HPA axis (Supplementary Table 1).

**Table 1:** Hormones of the HPA axis: mean difference from baseline

Parameter [Reference Range]/Time point/Difference from baseline	Placebo *N *= 17	2.5 mg *N *= 6	5 mg *N *= 6	10 mg *N *= 6	20 mg *N *= 6	40 mg *N *= 6	80 mg *N *= 6	160 mg *N *= 6	240 mg *N *= 6	360 mg *N *= 6
**Cortisol [171 – 536 nmol/L]**										
Baseline	396.1	359.7	338.1	395.6	330.8	301.3	325.7	258.1	219.4	347.8
24:00	-39.0	-77.1	1.8	-78.7	18.4	-48.8	102.6	-49.2	-7.3	-40.0
96:00	-46.6	-65.3	-75.0	-128.8	-34.1	-75.5	-64.4	-69.9	-5.1	-132.9
**ACTH [1.03 – 10.74 pmol/L]**										
Baseline	4.96	3.80	4.26	4.59	3.25	2.52	2.01	2.82	3.64	3.42
24:00	-1.22	0.59	0.53	0.78	1.53	1.44	4.69	1.34	0.87	1.30
96:00	-1.84	-0.46	-1.65	-1.35	-0.58	-0.01	1.36	0.05	-0.27	0.38
**DHEA-s [2.6 – 13.0 μmol/L]**										
Baseline	6.4	5.2	5.0	5.1	5.8	5.7	6.0	4.1	5.2	4.3
24:00	0.0	0.7	1.6	1.2	2.2	1.7	2.6	1.6	1.6	1.2
96:00	1.1	2.1	0.9	1.9	2.1	2.3	2.4	2.2	2.3	1.6
**Androstenedione [1.1 – 10.9 nmol/L]**										
Baseline	9.6	11.0	10.5	8.1	5.9	8.4	9.8	6.2	7.5	6.7
24:00	-2.3	0.2	1.2	1.3	3.7	3.7	8.1	2.6	5.0	3.2
96:00	-2.6	-1.8	-3.7	-3.4	3.8	0.1	0.5	1.5	2.7	-0.3
**Total Testosterone [9.9 – 27.9 nmol/L]**										
Baseline	16.1	18.4	16.7	18.7	15.4	20.3	16.3	13.4	15.6	11.6
24:00	0.2	0.1	-0.9	-0.2	-1.3	-0.5	0.8	1.0	-2.8	-0.3
96:00	-0.3	-0.3	-0.7	-2.0	-1.0	-0.8	0.6	2.4	-2.4	0.7
**SHBG [10 – 80 nmol/L]**										
Baseline	25.7	32.1	28.2	33.7	28.4	37.6	29.1	29.5	27.2	28.5
24:00	1.8	2.6	3.0	3.4	2.9	0.6	2.1	1.9	2.3	0.9
96:00	0.6	-0.1	2.7	0.2	1.3	-1.8	-1.3	-0.8	2.5	-2.2

### Pharmacokinetics

**Fig. 1:**
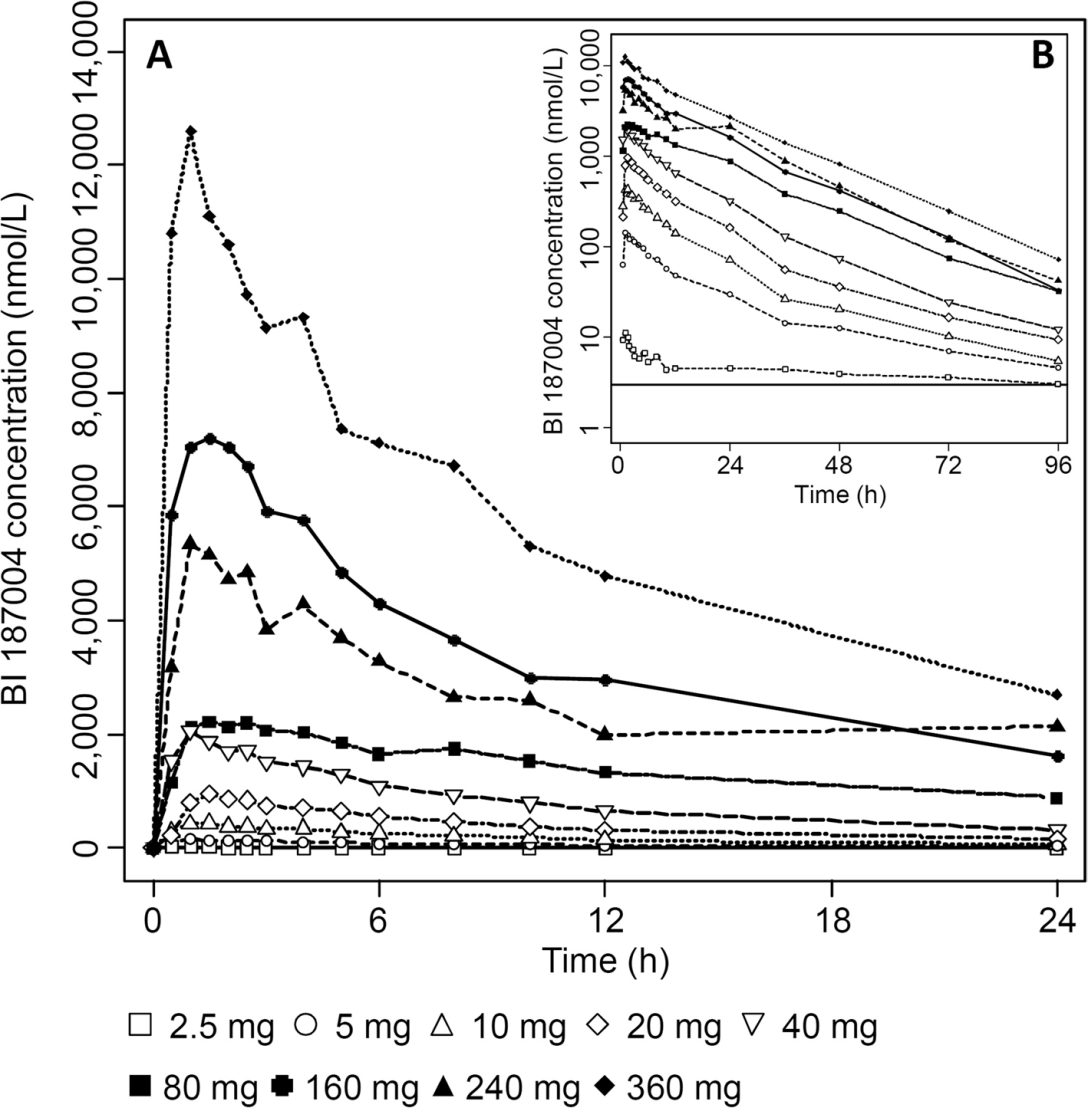
Arithmetic mean plasma concentration–time profile of BI 187004 after single oral administration of 2.5, 5, 10, 20, 40, 80, 160, 240 and 360 mg BI 187004 for (A) 24 h and (B) 96 h after drug administration. *n *= 5 (2.5 mg), *n *= 6 (5–360 mg)

In a few subjects from the 2.5 mg BI 187004 dose group, plasma concentrations were too low to estimate accurately the terminal half-life (t_1/2_) of BI 187004. For these subjects, several PK parameters like AUC_0-∞_, oral clearance (CL/F) or apparent volume of distribution (Vz/F) were not calculated. Dose normalized C_max_ and AUC values suggest a non-proportional increase in BI 187004 plasma exposure over the 2.5 to 360 mg dose range tested. A large inter-individual variability was noticeable for some dose groups investigated in this study. For the dose range of 5–360 mg regarding plasma exposure (C_max_/AUC), dose-proportionality can be assumed but not for elimination (Ae_0-72_). Plasma concentration–time profiles were generally characterized by a quick absorption after single oral administration of BI 187004 with peak concentrations observed between 1 h and 1.5 h after dosing, individual BI 187004 plasma concentrations are presented in Supplementary Tables 4–12. For a single subject (subject 64), C_max_ was reached 24 h after administration of 240 mg BI 187004. However, for this subject, plasma concentrations during the first 24 h were much lower compared to any subjects treated at this dose level (Supplementary Table 11). Thus, the plasma concentration time profile curve of the 240 mg group was substantially lower than the curve of the 160 mg group. The apparent clearance (CL/F) of BI 187004 was generally low (4.21—6.44 L/h). A trend showing a continuous decrease in CL/F values from 5 to 160 mg BI 187004 and then an increase for doses of 240 and 360 mg was observed. The volume of distribution (V_z_/F) of BI 187004 was moderate (87.9—281.0 L) and a similar trend as for CL/F was observed across the BI 187004 tested dose range. The apparent terminal t_1/2_ of BI 187004 decreased from 33.5 h to 14.5 h between the 5 mg and 160 mg dose groups. At higher BI 187004 doses (240, 360 mg) t_1/2_ was comparable to the 160 mg group. The cumulative fraction of the BI 187004 dose excreted in urine during the 0–72 h collection interval was low (3.00–4.93%) with the largest proportion being excreted during the first 12 h post dosing. The data are summarized in Table 2 and Fig. 1.

**Table 2:** Summary of pharmacokinetic parameters of BI 187004 after single oral dose administrations in healthy male volunteers

Pharmacokinetic parameter	BI 187004								
	**2.5 mg** **gMean (%gCV)**	**5 mg** **gMean (%gCV)**	**10 mg** **gMean (%gCV)**	**20 mg** **gMean (%gCV)**	**40 mg** **gMean (%gCV)**	**80 mg** **gMean (%gCV)**	**160 mg** **gMean (%gCV)**	**240 mg** **gMean (%gCV)**	**360 mg** **gMean (%gCV)**
AUC_0-∞_ [nmol*h/L]	– (–)	2,500 (23.0)	5,910 (29.2)	12,400 (41.2)	25,900 (36.1)	51,100 (40.2)	111,000 (19.5)	109,000 (53.4)	191,000 (25.4)
AUC_0-24_ [nmol*h/L]	212 (35.2)	1,490 (21.5)	4,380 (23.2)	9,450 (32.1)	19,900 (26.6)	43,100 (36.9)	82,100 (12.6)	71,700 (74.1)	135,000 (22.0)
C_max_ [nmol/L]	11.8 (75.0)	150 (19.7)	502 (14.0)	1,030 (14.4)	2,210 (27.8)	2,460 (56.2)	8,310 (11.0)	7,120 (64.9)	13,400 (34.1)
C_24_ [nmol/L]	4.54 (25.2)	30.0 (28.0)	71.3 (46.1)	161 (66.3)	321 (50.8)	878 (36.0)	1,630 (25.9)	2,130 (22.5)	2,690 (36.0)
t_max_ [h]*	1.0 (1.0—1.5)	1.0 (1.0—1.5)	1.0 (0.5—2.5)	1.38 (0.5—1.5)	1.0 (0.5—3.0)	2.25 (1.0—2.5)	1.0 (0.5—2.0)	1.0 (1.0—24.0)	1.0 (0.5—1.0)
t_1/2_ [h]	–	33.5 (14.4)	23.7 (18.0)	21.2 (17.8)	18.9 (19.7)	17.1 (35.6)	14.5 (8.92)	13.4 (28.9)	13.9 (16.0)
V_Z_/F [L]	–	281 (27.7)	169 (44.3)	144 (52.2)	123 (49.8)	113 (53.3)	87.9 (14.6)	124 (48.2)	110 (16.1)
CL/F [L/h]	–	5.82 (23.0)	4.93 (29.2)	4.70 (41.2)	4.50 (36.1)	4.56 (40.2)	4.21 (19.5)	6.44 (53.4)	5.48 (25.4)
fe_0-72 h_ [%]	–	3.00	4.03	4.93	4.21	4.55	4.75	4.88	4.25

– no descriptive statistics calculated, *gMean* Geometric mean, *%gCV* Geometric coefficient of variation, *AUC*_0-∞_ Area under the concentration–time curve over time interval from 0, extrapolated to infinity, *AUC*_0-24_ Area under the concentration vs time curve during one dosing interval, *C*_max_ Maximum observed drug concentration, *C*_24_ Drug concentration in 24 h dosing interval, *t*_max_ Time of maximum observed drug concentration, *t_max_ Is given as median and range (min – max), *t*_1/2_ Half-life associated with terminal rate constant in non-compartmental analysis, *VZ/F* Apparent volume of distribution during the terminal phase λz, *CL/F* Oral clearance, fe0-72 h Fraction of BI 187004 eliminated in urine from 0 to 72 h

BI 187004 concentration in plasma compared with the concentration in AT at 24 h post dosing, was approximately 10 times higher (range of ratio AT_C24_ /Plasma_C24_: 8.58—11.4). Plasma concentration at 10 h post dosing compared to the AT concentrations showed factor between 8.36 and 16.2 times higher values except for the 240 mg dose group in which the gMean ratio plasma concentration /AT concentration was higher (25.1%) due to very low BI 187004 plasma concentrations observed in one subject (Table 3).

**Table 3:** PK/PD relationship between BI 187004 concentration in adipose tissue and plasma and ex vivo 11beta-HSD1 inhibition in AT after a single dose of BI 187004

Dose (mg)	BI 187004 Plasma C_10_ gMean (range) [nmol/L]	BI 187004 AT C_10_ gMean (range) [nmol/L]	BI 187004 AT:plasma ratio (range) 10 h	11beta-HSD1 inhibition in AT* median (min–max) 10 h [%]	BI 187004 Plasma C_24_ gMean (range) [nmol/L]	BI 187004 AT C_24_ gMean (range) [nmol/L]	BI 187004 AT:plasma ratio (range) 24 h	11beta-HSD1 inhibition in AT* median (min–max) 24 h [%]
10	174 (118 – 250)	17.9 (12.3 – 26.7)	10.3 (7.78 – 14.8)	86.8 (81.1 – 91.4)	71.3 (39.1 – 112)	– (9.91 – 22.6)	– (9.58 – 20.1)	59.4 (34.0 – 78.6)
20	382 (253 – 529)	38.1 (26.2 – 49.5)	9.97 (8.47 – 13.4)	81.5 (68.5 – 93.6)	161 (85.8 – 353)	17.5 (11.1 – 33.3)	10.8 (5.90 – 16.7)	82.3 (65.0 – 90.7)
40	796 (467 – 1,140)	80.3 (67.6 – 122)	10.1 (7.22 – 14.5)	95.6 (93.6 – 96.7)	321 (169 – 625)	27.5 (18.9 – 42.7)	8.58 (6.84 – 12.5)	84.6 (82.9 – 95.0)
80	1,530 (1,040 – 2,570)	128 (92.3 – 207)	8.36 (6.71 – 12.2)	97.5 (95.7 – 98.8)	878 (605 – 1,560)	84.5 (63.7 – 128)	9.62 (8.18 – 12.0)	93.5 (91.1 – 96.9)
160	3,000 (1,940 – 4,290)	412 (328 – 58 2)	13.7 (8.74 – 20.7)	99.1 (98.8 – 99.4)	1,630 (1,360 – 2,690)	168 (105 – 325)	10.3 (7.73 – 12.4)	97.9 (94.0 – 98.9)
240	2,600 (313 – 5,800)	653 (474 – 899)	25.1 (11.4 – 207)	99.4 (99.1 – 99.6)	2,130 (1,530 – 2,630)	212 (143 – 283)	9.96 (8.32 – 11.7)	98.1 (96.8 – 98.8)
360	5,300 (3,360 – 7,680)	857 (595 – 1,480)	16.2 (9.31 – 34.4)	99.5 (99.3 – 99.6)	2,690 (1,790 – 4,740)	307 (191 – 404)	11.4 (8.51 – 15.0)	98.6 (98.0 – 99.4)

– no descriptive statistics calculated (> 60% of subjects had concentrations below lower limit of quantification), *gMean* Geometric mean, *AT* Adipose tissue, *C*_10_ Drug concentration 10 h after single dose treatment, *C*_24_ Drug concentration 24 h after single dose treatment

* 11beta-HSD1 inhibition in AT defined as % decrease from baseline in d2 cortisol levels

### 11beta-HSD1 inhibition in the liver

**Fig. 2:**
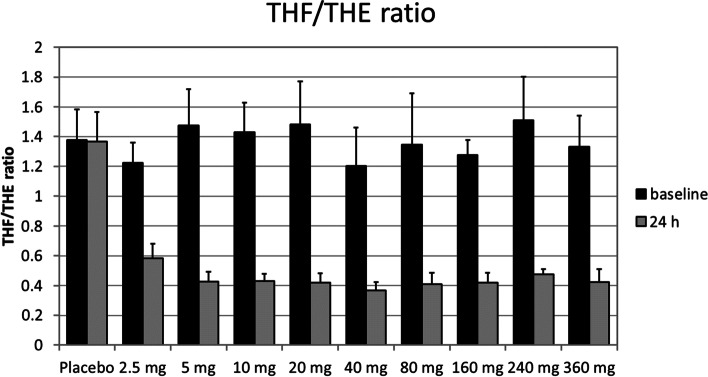
Tetrahydrocortisol (aTHF + THF) / tetrahydrocortisone (THE) at baseline and after single ascending dose administrations in healthy volunteers. Data are mean ± SD, *n *= 14 (placebo), *n *= 6 (BI 187004)

The pharmacodynamic effects of BI 187004 were evaluated based on the urinary concentrations of cortisone (UFE, UTE), cortisol (UFF, UTF) and their respective metabolites (aTHF, THF, THE) over a 24 h period. Inhibition of 11beta-HSD1 in the liver was assessed indirectly by urinary concentration of THF and THE, i.e. the ratio of (aTHF + THF)/THE (in short: (aTHF + THF)/THE ratio), by calculating the change from baseline related to baseline and compared for treatment with single ascending doses of BI 187004 versus placebo (placebo-corrected change from baseline of the (aTHF + THF)/THE). Inhibition in the 2.5 mg group was calculated as 54.8% and by 69.9% in the 5 mg group remaining on this level in all higher treatment groups (10–360 mg) without any dose-dependent effect (Fig. 2) Urinary THF/THE ratios are summarized in Supplementary Table 2. Individual THF/THE ratios are presented in Supplementary Table 13.

Furthermore, total urinary corticosteroid excretion (sum of UFF, UFE, aTHF, THF and THE) increased up to 197.5% indicating activation of cortisol turnover (Supplementary Table 1).

### 11beta-HSD1 inhibition in adipose tissue

**Fig. 3:**
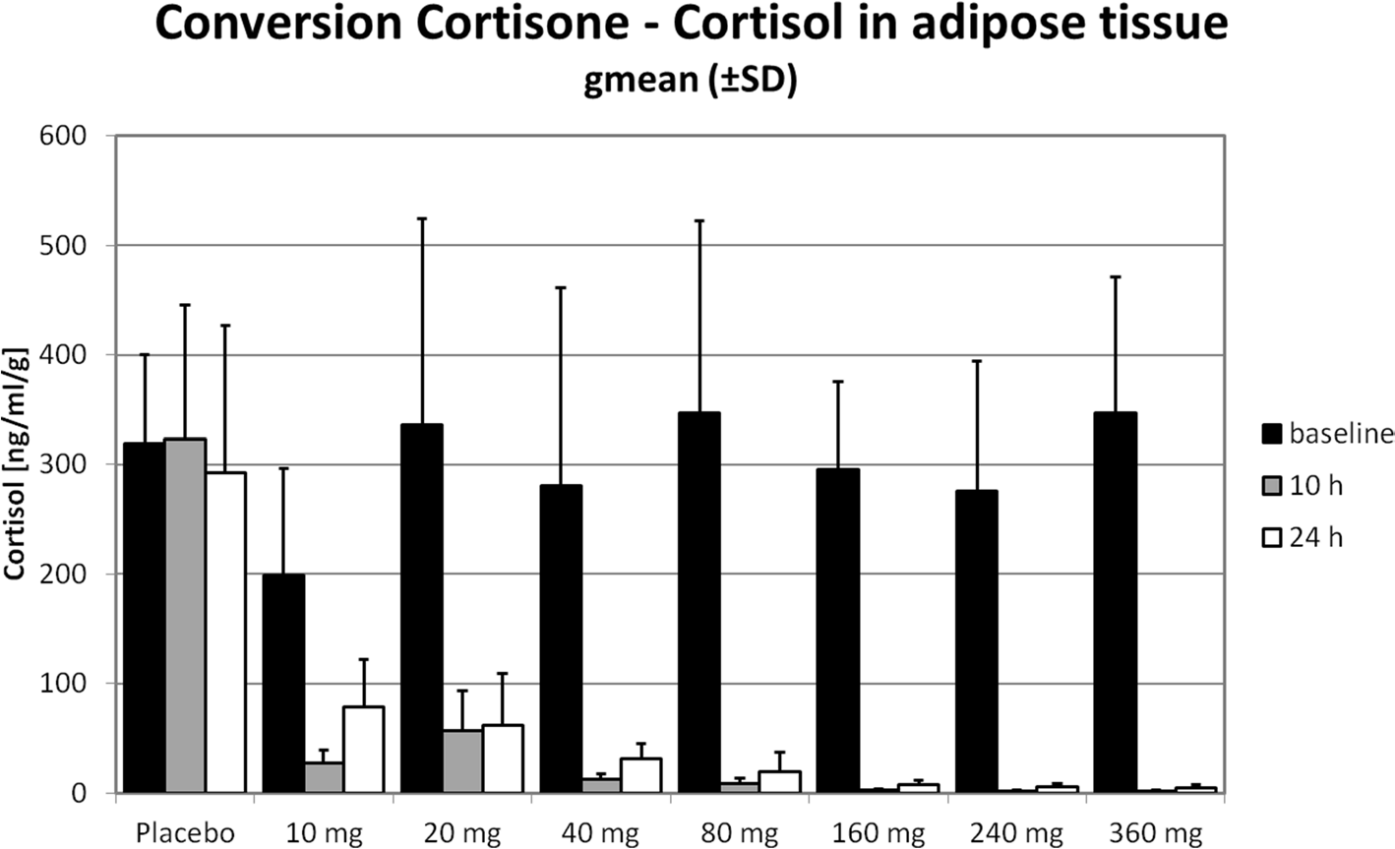
11beta-HSD1 inhibition in adipose tissue 10 h and 24 h after single oral doses of BI 187004 in healthy male volunteers. Data are median ± SD, *n *= 14 (placebo), *n *= 6 (BI 187004)

Conversion of d2-cortisone to d2-cortisol in AT as measure of 11beta-HSD1 enzyme activity 10 h after a single dose of BI 187004 decreased by 86.8% in the 10 mg and by 81.5% in the 20 mg dose group and remained greater than 95% in the dose groups 40–360 mg. 24 h after a single dose cortisone to cortisol conversion was 59.4—84.6% in the dose groups 10–40 mg and remained greater than 95% in the dose groups 80–360 mg (Table 3, Fig. 3). Individual cortisol levels are presented in Supplementary Table 14.

## Discussion

The results of this randomized, double-blind, placebo-controlled, single rising dose study are the first clinical data from the 11beta-HSD1 inhibitor BI 187004 in humans. The compound in single doses of up to 360 mg was well tolerated in healthy male subjects. The overall incidence of AEs was low, did not show any dose-dependency and was not different between BI 187004-treated and placebo-treated subjects.

BI 187004 was generally absorbed quickly after the administration of the oral solution with a non-proportional increase in BI 187004 plasma exposure over the entire dose range. The plasma PK profiles observed showed a change in distribution and elimination of BI 187004 over the entire dose range of 2.5 to 360 mg BI 187004: From 2.5 to 160 mg volume of distribution as well as clearance showed a trend to decrease and then increased for the doses 240 and 360 mg as possible explanation of these changes. However, a possible increase in the absorption of BI 187004 could also explain the change in distribution and clearance.

The decrease in the plasma terminal half-life of BI 187004 may be explained by a smaller decrease in the oral clearance compared to the apparent volume of distribution but can also be related to the lack of PK samples collected beyond 96 h to determine the actual terminal phase for BI 187004 doses above 2.5 mg. In the 240 mg dose group a single subject reached C_max_ only 24 h after drug administration and showed plasma concentrations which were approximately tenfold to 60-fold lower compared to the measurements of the other subjects at the individual time points. Thus, the plasma concentration time profile curve of the 240 mg is substantially lower than the curve of the 160 mg dose group.

Of particular interest for this mode of action are changes in the plasma hormone concentrations as summarized by Stomby et al. [13]. Extra-adrenal regeneration of cortisol by 11beta-HSD1 substantially contributes to the overall amount of circulating cortisol. Successful 11beta-HSD1 enzyme inhibition is expected to enhance the net metabolic clearance rate of cortisol. Thus, in response to 11beta-HSD1 inhibition serum cortisol levels tend to decrease but due to loss of negative HPA axis feedback there occurs a compensatory increase in ACTH and the cortisol secretion rate. Furthermore, DHEA-s and androstenedione levels are expected to increase while serum cortisol overall remains stable or slightly decreases. In line with the findings of Stomby [13] our data also suggest that a single dose of an 11beta-HSD1 inhibitor is likely not sufficient to significantly activate the HPA axis: after single ascending doses of BI 187004, all HPA axis hormones remained within the normal range. However, there was a transient increase of ACTH 24 h after the single dose of BI 187004. Also total urinary corticosteroid excretion increased by up to twofold, which indicates a transient activation of the HPA axis hormones.

Data from mice indicate that wild type mice mostly excrete urinary corticosteroids as metabolites of corticosterone and less than 10% as 11-Dehydrocorticosterone (11-DHC) metabolites, whereas in whole body 11beta-HSD1 knockout mice, 20–25% are excreted as 11-DHC metabolites [14]. In contrast, liver specific 11beta-HSD knockout mice showed similar glucocorticoid excretion patterns as control mice [15]. Thus, at least in animal models, urinary steroid metabolite excretion might be substantially influenced by extrahepatic inhibition of 11beta-HSD1. In clinical studies, reduction in urinary (aTHF + THF)/THE ratio is still used as relevant non-invasive indirect measure of 11beta-HSD1 enzyme inhibition in the liver [16]. In our study urinary (aTHF + THF)/THE ratio decreased by approximately 70% after a single dose of 5 mg BI 187004 with no further decrease in all higher dose groups. Only the lowest dose of 2.5 mg showed a slightly less decrease in the (aTHF + THF)/THE ratio by 54.8%. Thus, a true dose–response relationship could not be established. In addition, there were only minimal changes over time suggesting the maximum hepatic 11beta-HSD1 inhibition for BI 187004 was already reached in the second lowest dose of 5 mg after single dosing.

A reduction in (aTHF + THF)/THE ratio by 70% after single dose treatment is in the range of what was previously reported for other 11beta-HSD1 inhibitors: maximum decrease in the urinary (aTHF + THF)/THE ratio was as high as 92% for RO5093151 after 4 weeks [17] and 92% at the highest dose of 900 mg for AZD4017 after 9 days [18] and as low as 26% for PF-915275 after 14 days of treatment [19]. In patients with T2DM treatment with BI 135585 decreased urinary (aTHF + THF)/THE ratio by 65–75% after a single dose and by 75% after 14 days compared to placebo which was already achieved in the lowest dose (5 mg) without further decrease with higher doses [20]. Currently, the question remains to what extent inhibition of 11beta-HSD1 in the liver leads to a reduction in the (aTHF + THF)/THE ratio. Although this open question remains, we demonstrated that BI 187004 led to a significant and sustained 11beta-HSD1 inhibition in the liver as indicated by the reduction in urinary (aTHF + THF)/THE ratio already at low doses.

Complementary analyses of 11beta-HSD1 enzyme inhibition in AT showed consistent results. Single ascending doses of 10–360 mg BI 187004 led to 11beta-HSD1 inhibition of  >90% 10 h after single dosing in the 40 mg group and 24 h after single dosing in the 80 mg group. The strong enzyme inhibition of >90% was expected based on the achieved BI 187004 concentration in AT and preclinical modeling data using the 11beta-HSD1 inhibitor BI 135585 [21].

The data are in line with published data with other 11beta-HSD1 inhibitors: Several groups reported data on 11beta-HSD1 inhibition in AT after single dose treatment. For instance, INCB13739 inhibited 11beta-HSD1 enzyme >90% in AT after a single dose for at least 24 h based on a decrease in (aTHF + THF)/THE ratio [22]. Similarly, single dose of AMG 221 administration resulted in sustained >90% 11beta-HSD1 inhibition in AT for the 24-h study duration [23]. Likewise, a single dose of BI 135585 led to 90% inhibition of 11beta-HSD1 in AT 24 h after dosing [20].

Based on preclinical modeling [21] an inhibition of >90% of 11beta-HSD1 in AT is regarded as a prerequisite to obtain sustained glucose lowering efficacy. Therefore, a dose of at least 40–80 mg BI 187004 is thought to be needed to achieve a long-term blood glucose following long-term effect.

In summary, BI 187004 leads to a significant and strong reduction in urinary (aTHF + THF)/THE ratio indicating 11beta-HSD1 inhibition in the liver after single rising doses as well as a dose dependent almost full inhibition of 11beta-HSD1 in AT. As expected, BI 187004 did transiently activate the HPA axis after single doses. In contrast to the clinical development of other 11beta-HSD1 inhibitors, data on enzyme inhibition in liver and AT were assessed with BI 187004 already in the first-in-human trial in subjects with overweight or obesity, a population close to the target population of T2DM and obesity.

This first-in-human trial demonstrated that BI 187004 is a potent 11beta-HSD1 inhibitor in both, liver and AT. Therefore, it fulfils the prerequisites to evaluate the effect of 11beta-HSD1 inhibition in improving glucose metabolism, body weight and lipid profile in patients with T2DM and obesity.

## Conclusion

It can be concluded that, (i) the novel 11beta-HSD1 inhibitor BI 187004 is safe and well tolerated after single dose in healthy male volunteers with overweight or obesity; (ii) there is significant and over 24 h sustained 11beta-HSD1 enzyme inhibition in liver and AT; (iii) the promising pharmacodynamic effects justify further investigation of BI 187004 in multiple dose trials.

## Supplementary Information

**Additional file 1: Supplementary Table 1. **Summary of total urinary corticosteroids (aTHF + THF + UFF + UFE + THE) by time intervals. **Supplementary Table 2. **Summary of urinary THF (5alpha-THF + 5beta-THF)/THE ratio by time intervals and by placebo-corrected, baseline-adjusted gmeans. **Supplementary Table 3. **Baseline metabolic parameters. **Supplementary Table 4. **Individual drug plasma concentration of BI 187004 after single oral administration of 2.5 mg BI 187004 with descriptive statistics. **Supplementary Table 5. **Individual drug plasma concentration of BI 187004 after single oral administration of 5 mg BI 187004 with descriptive statistics. **Supplementary Table 6. **Individual drug plasma concentration of BI 187004 after single oral administration of 10 mg BI 187004 with descriptive statistics. **Supplementary Table 7. **Individual drug plasma concentration of BI 187004 after single oral administration of 20 mg BI 187004 with descriptive statistics. **Supplementary Table 8. **Individual drug plasma concentration of BI 187004 after single oral administration of 40 mg BI 187004 with descriptive statistics. **Supplementary Table 9. **Individual drug plasma concentration of BI 187004 after single oral administration of 80 mg BI 187004 with descriptive statistics. **Supplementary Table 10. **Individual drug plasma concentration of BI 187004 after single oral administration of 160 mg BI 187004 with descriptive statistics. **Supplementary Table 11. **Individual drug plasma concentration of BI 187004 after single oral administration of 240 mg BI 187004 with descriptive statistics. **Supplementary Table 12. **Individual drug plasma concentration of BI 187004 after single oral administration of 360 mg BI 187004 with descriptive statistics. **Supplementary Table 13. **Individual THF/THE ratio at baseline and after single ascending dose administrations in healthy volunteers. **Supplementary Table 14. **Individual cortisol levels in adipose tissue at baseline, 10 h and 24 h after single ascending dose administrations in healthy volunteers.

## Data Availability

The datasets used and/or analyzed during the current study are available from the corresponding author on reasonable request.

## Abbreviations

%gCV: Geometric coefficient of variation
μg: Microgram
11beta-HSD1: 11beta-hydroxysteroid dehydrogenase-1
11-DHC: 11-dehydrocortisol
aTHF: 5alpha-tetrahydrocortisol
THF: 5beta-tetrahydrocortisol
ACTH: Adrenocorticotropic hormone
AE: Adverse event
Ae: Amount of drug excreted into urine
AT: Adipose tissue
AUC: Area under the curve
AUC_0-24_: Area under the concentration vs time curve during one dosing interval
AUC_0-∞_: Area under the concentration–time curve over time interval from 0 extrapolated to infinity
BMI: Body mass index
C_10_: Drug concentration 10 h after single dose treatment
C_24_: Drug concentration 24 h after single dose treatment
CI: Confidence interval
CL/F: Drug clearance from plasma after oral administration
C_max_: Maximum observed drug concentration in plasma
D2: Deuterized
DHEA-s: Dehydroepiandrosterone-sulphate
ECG: Electrocardiogram
fe_0-72__ h_: Fraction of BI 187004 eliminated in urine from 0 to 72 h
gMean: Geometric mean
h: Hour
HPA: Hypothalamus–pituitary–adrenal
L: Liter
LC–MS/MS: Liquid chromatography-tandem mass spectrometry
mg: Milligram
PD: Pharmacodynamics
PFOS: Powder for oral solution
PK: Pharmacokinetics
RE: Relative error
RSD: Relative standard deviation
SD: Standard deviation
SHBG: Sex-hormone binding globulin
t_1/2_: Half-life
T2DM: Type 2 diabetes mellitus
THE: Tetrahydrocortisone
t_max_: Time of maximum observed drug concentration
UFE: Urinary free cortisone
UFF: Urinary free cortisol
UTE: Urinary total cortisone
UTF: Urinary total cortisol
V_z_/F: Apparent volume of distribution
VZ/F: Apparent volume of distribution during the terminal phase λz
