# Supplementary material for: Safety, tolerability, pharmacokinetics and pharmacodynamics of single oral doses of BI 187004, an inhibitor of 11beta-hydroxysteroid dehydrogenase-1, in healthy male volunteers with overweight or obesity
Source: Clin Diabetes Endocrinol. 2021 Aug 15;7:16. doi: 10.1186/s40842-021-00130-x (PMC8364686; doi:10.1186/s40842-021-00130-x)
Supplement: Supplementary file 1 — Additional file 1: Supplementary Table 1. Summary of total urinary corticosteroids (aTHF + THF + UFF + UFE + THE) by time intervals. Supplementary Table 2. Summary of urinary THF (5alpha-THF + 5beta-THF)/THE ratio by time intervals and by placebo-corrected, baseline-adjusted gmeans. Supplementary Table 3. Baseline metabolic parameters. Supplementary Table 4. Individual drug plasma concentration of BI 187004 after single oral administration of 2.5 mg BI 187004 with descriptive statistics. Supplementary Table 5. Individual drug plasma concentration of BI 187004 after single oral administration of 5 mg BI 187004 with descriptive statistics. Supplementary Table 6. Individual drug plasma concentration of BI 187004 after single oral administration of 10 mg BI 187004 with descriptive statistics. Supplementary Table 7. Individual drug plasma concentration of BI 187004 after single oral administration of 20 mg BI 187004 with descriptive statistics. Supplementary Table 8. Individual drug plasma concentration of BI 187004 after single oral administration of 40 mg BI 187004 with descriptive statistics. Supplementary Table 9. Individual drug plasma concentration of BI 187004 after single oral administration of 80 mg BI 187004 with descriptive statistics. Supplementary Table 10. Individual drug plasma concentration of BI 187004 after single oral administration of 160 mg BI 187004 with descriptive statistics. Supplementary Table 11. Individual drug plasma concentration of BI 187004 after single oral administration of 240 mg BI 187004 with descriptive statistics. Supplementary Table 12. Individual drug plasma concentration of BI 187004 after single oral administration of 360 mg BI 187004 with descriptive statistics. Supplementary Table 13. Individual THF/THE ratio at baseline and after single ascending dose administrations in healthy volunteers. Supplementary Table 14. Individual cortisol levels in adipose tissue at baseline, 10 h and 24 h after single ascending dose administration [file 40842_2021_130_MOESM1_ESM.docx]

**Supplementary Table 1:**

Summary of total urinary corticosteroids (aTHF + THF + UFF + UFE + THE) by time intervals

| BI 187004 | Total corticosteroids [μg] | | |  | |
| --- | --- | --- | --- | --- | --- |
|  | **Descriptive analysis**  **Comparison of post-dosing versus baseline values** | | | **ANCOVA based analysis**  **Estimation of placebo-corrected adjusted gMeans** | |
| **Dose Group** | **-24 – 0h (day -1)**  **gMean gCV [%]** | **0 – 24h (day 1)**  **gMean gCV [%]** | **% of baseline***  **0 – 24h (day 1)/**  **-24 – 0h (day -1)**  **gMean gCV [%]** | **Placebo corrected % of baseline***  **0 – 24h (day 1) /-24 – 0h (day -1) adjusted gMean (90% CI) [%]** | **Placebo corrected % change from baseline****  **Adjusted gMean (90% CI) [%]**  **p-value for change=0** |
| 2.5 mg (N=6) | 9957.93  (53.522) | 16067.0  (18.128) | 161.3  (44.4) | 130.3  (111.5, 152.2) | 30.3 (11.5, 52.2)  0.0062 |
| 5 mg (N=6) | 13519.1  (31.832) | 24324.0  (23.620) | 179.9  (21.8) | 172.1  (147.7, 200.6) | 72.1 (47.7, 100.6)  <0.0001 |
| 10 mg (N=6) | 13453.5  (23.037) | 23965.4  (24.142) | 178.1  (24.5) | 169.9  (145.8, 198.1) | 69.9 (45.8, 98.1)  <0.0001 |
| 20 mg (N=6) | 15589.7  (32.177) | 27240.6  (5.276) | 174.7  (31.3) | 180.9  (154.9, 211.2) | 80.9 (54.9, 111.2)  <0.0001 |
| 40 mg (N=6) | 11446.7  (16.080) | 24484.7  (17.661) | 213.9  (24.4) | 186.6  (160.0, 217.5) | 86.6 (60.0, 117.5)  <0.0001 |
| 80 mg (N=6) | 15751.4  (30.962) | 29884.0  (24.608) | 189.7  (16.0) | 197.5  (169.1, 230.7) | 97.5 (69.1, 130.7)  <0.0001 |
| 160 mg (N=6) | 14780.4  (27.246) | 25409.0  (28.223) | 171.9  (13.1) | 172.8  (148.1, 201.6) | 72.8 (48.1, 101.6)  <0.0001 |
| 240 mg (N=6) | 15398.0  (31.137) | 26872.4  (19.594) | 174.5  (17.4) | 179.4  (153.7, 209.5) | 79.4 (53.7, 109.5)  <0.0001 |
| 360 mg (N=6) | 15564.1  (28.661) | 26152.2  (30.046) | 168.0  (28.0) | 173.8  (148.8, 202.9) | 73.8 (48.8, 102.9)  <0.0001 |
| **Placebo (N=17)** | **12587.8**  **(42.126)** | **13691.4**  **30.453** | **108.8**  **(30.6)** | **-** | **-** |

* % of baseline: ratio of values post-dose over baseline [%]

** % change from baseline: ratio change from baseline over baseline [%]

**Supplementary Table 2:**

Summary of urinary THF (5alpha-THF + 5beta-THF)/THE ratio by time intervals and by placebo-corrected, baseline-adjusted gmeans

|  |  | | |  | |
| --- | --- | --- | --- | --- | --- |
| **BI 187004** | **(5alpha-THF + 5beta-THF)/THE** | | | | |
|  | **Descriptive analysis**  **Comparison of post-dosing versus baseline values [%] per treatment** | | | **ANCOVA based analysis**  **Estimation of placebo-corrected adjusted gMeans** | |
| **Dose Group** | **-24 – 0h (day -1)**  **gMean gCV [%]** | **0 – 24h (day 1)**  **gMean gCV [%]** | **% of baseline*  0 – 24h (day 1) /**  **-24 – 0h (day -1)**  **gMean gCV [%]** | **Placebo corrected % of baseline***  **0 – 24h (day 1) /-24 – 0h (day -1) adjusted gMean (90% CI) [%]** | **Placebo corrected % change from baseline****  **Adjusted gMean (90% CI) [%]**  **p-value for change=0** |
| **2.5 mg (N=6)** | 1.224  10.949 | 0.584  16.940 | 47.7  20.5 | 45.2  (40.8, 50.1) | -54.8 (-59.2, -49.9)  <0.0001 |
| **5 mg (N=6)** | 1.476  17.563 | 0.426  16.039 | 28.9  25.4 | 30.1  (27.2, 33.4) | -69.9 (-72.8, -66.6)  <0.0001 |
| **10 mg (N=6)** | 1.429  13.389 | 0.430  10.966 | 30.1  21.5 | 30.9  (27.9, 34.2) | -69.1 (-72.1, -65.8)  <0.0001 |
| **20 mg (N=6)** | 1.481  19.014 | 0.421  13.928 | 28.4  10.7 | 29.7  (26.8, 32.9) | -70.3 (-73.2, -67.1)  <0.0001 |
| **40 mg (N=6)** | 1.204  23.476 | 0.367  14.669 | 30.5  15.2 | 28.7  (25.8, 31.8) | -71.3 (-74.2, -68.2)  <0.0001 |
| **80 mg (N=6)** | 1.345  25.128 | 0.410  17.837 | 30.5  13.7 | 30.3  (27.4, 33.6) | -69.7 (-72.6, -66.4)  <0.0001 |
| **160 mg (N=6)** | 1.275  7.713 | 0.419  17.179 | 32.8  17.8 | 31.8  (28.7, 35.2) | -68.2 (-71.3, -64.8)  <0.0001 |
| **240 mg (N=6)** | 1.510  18.193 | 0.475  7.359 | 31.5  12.8 | 33.3  (30.0, 36.8) | -66.7 (-70.0, -63.2)  <0.0001 |
| **360 mg (N=6)** | 1.333  15.147 | 0.424  19.479 | 31.8  13.7 | 31.5  (28.5, 34.9) | -68.5 (-71.5, -65.1)  <0.0001 |
| **Placebo (N=17)** | 1.377  14.479 | 1.367  14.217 | 99.3  7.7 |  |  |
|  |  |  |  | **-** | **-** |

* % of baseline: ratio of values post-dose over baseline [%]

** % change from baseline: ratio change from baseline over baseline [%]

**Supplementary Table 3**

Baseline metabolic parameters

| **Parameter [Unit]** | **Placebo**  **N=17**  **Mean**  **(min, max)** | **2.5 mg**  **N=6**  **Mean**  **(min, max)** | **5 mg**  **N=6**  **Mean**  **(min, max)** | **10 mg**  **N=6**  **Mean**  **(min, max)** | **20 mg**  **N=6**  **Mean**  **(min, max)** | **40 mg**  **N=6**  **Mean**  **(min, max)** | **80 mg**  **N=6**  **Mean**  **(min, max)** | **160 mg**  **N=6**  **Mean**  **(min, max)** | **240mg**  **N=6**  **Mean**  **(min, max)** | **360 mg**  **N=6**  **Mean**  **(min, max)** |
| --- | --- | --- | --- | --- | --- | --- | --- | --- | --- | --- |
| Fasting plasma glucose [mmol/l] | 5.5  (4.9, 7.0) | 5.4  (5.2, 5.7) | 5.4  (5.1, 6.0) | 5.6  (4.8, 6.3) | 5.5  (5.0, 6.0) | 5.8  (5.2, 6.3) | 5.9  (5.4, 6.3) | 5.6  (5.3, 6.1) | 5.8  (5.3, 6.7) | 5.9  (5.5, 6.2) |
| LDL Cholesterol [mmol/l] | 2.4  (1.5, 3.8) | 2.3  (1.6, 3.5) | 2.7  (1.6, 3.7) | 2.3  (1.5, 4.2) | 2.5  (1.9, 3.7) | 2.6  (2.0, 3.9) | 2.3  (1.8, 3.1) | 3.2  (2.3, 4.0) | 2.4  (1.8, 2.9) | 2.8  (2.6, 3.2) |
| Waist to hip ratio | 1.00  (0.9, 1.1) | 1.02  (1.0, 1.1) | 1.01  (1.0, 1.0) | 0.98  (0.9, 1.1) | 0.98  (0.9, 1.1) | 1.06  (1.0, 1.1) | 1.01  (0.9, 1.1) | 1.01  (1.0, 1.1) | 1.02  (0.9, 1.1) | 1.05  (0.9, 1.1) |
| Total body mass [kg] | 98.4  (80.9, 126.6) | 87.8  (76.8, 92.7) | 92.9  (72.8, 106.4) | 97.2  (79.8, 110.5) | 103.3  (94.6, 118.0) | 100.7  (77.4, 127.5) | 102.7  (90.8, 113.9) | 98.5  (87.9, 105.8) | 109.1  (87.7, 131.7) | 107.9  (89.9, 133.1) |
| Body fat [%] | 30.5  (8.2, 42.8) | 27.2  (17.2, 38.2) | 31.2  (26.1, 43.3) | 31.9  (25.3, 35.1) | 30.7  (25.6, 37.4) | 35.9  (27.3, 39.9) | 33.7  (22.8, 41.3) | 32.2  (18.4, 39.4) | 36.9  (24.9, 42.5) | 38.8  (28.0, 45.6) |
| Systolic Blood pressure [mmHg] | 126  (107, 147) | 121  (118, 128) | 118  (107, 126) | 122  (114, 135) | 130  (118, 150) | 124  (113, 142) | 131  (124, 143) | 125  (116, 140) | 129  (112, 139) | 130  (113, 146) |
| Diastolic blood pressure [mmHg] | 76  (62, 99) | 74  (64, 90) | 75  (65, 91) | 79  (74, 87) | 88  (80, 98) | 83  (71, 95) | 81  (70, 91) | 81  (70, 92) | 84  (79, 89) | 85  (66, 98) |
| Pulse rate [bpm] | 58  (45, 73) | 61  (53, 70) | 57  (44, 76) | 56  (44, 69) | 56  (45, 66) | 58  (49, 66) | 62  (45, 68) | 57  (46, 66) | 60  (50, 74) | 56  (46, 64) |

**Supplementary Table 4:**

Individual drug plasma concentration of BI 187004 after single oral administration of 2.5 mg BI 187004 with descriptive statistics

| 2.5 mg  BI 187004 | BI 187004 plasma concentrations [nmol/L]  Planned times [h] | | | | | | | | | | | | | | | | | |
| --- | --- | --- | --- | --- | --- | --- | --- | --- | --- | --- | --- | --- | --- | --- | --- | --- | --- | --- |
| **Subject** | **–0.5** | **0.5** | **1** | **1.5** | **2** | **2.5** | **3** | **4** | **5** | **6** | **8** | **10** | **12** | **24** | **36** | **48** | **72** | **96** |
|  |  |  |  |  |  |  |  |  |  |  |  |  |  |  |  |  |  |  |
| **2** | BLQ | 5.84 | 6.77 | 6.06 | 4.97 | 4.45 | 3.48 | 3.40 | BLQ | BLQ | BLQ | BLQ | BLQ | 4.19 | BLQ | 3.58 | BLQ | BLQ |
| **3** | BLQ | 15.8 | 28.4 | 17.2 | 13.8 | 12.5 | 11.9 | 10.7 | 9.77 | 7.52 | 6.73 | 5.99 | 6.44 | 6.66 | 5.06 | 5.01 | 3.34 | BLQ |
| **4** | BLQ | 8.84 | 17.6 | 14.9 | 11.0 | 10.1 | 8.37 | 7.79 | 7.70 | 6.22 | 6.55 | 5.19 | 4.76 | 5.03 | 3.88 | 4.22 | 3.91 | 3.04 |
| **5** | BLQ | BLQ | 7.86 | 12.1 | 9.41 | 8.45 | 6.66 | 6.03 | 6.49 | 5.34 | 5.06 | 3.85 | 4.10 | 3.69 | BLQ | BLQ | BLQ | BLQ |
| **7** | BLQ | BLQ | 5.64 | 5.62 | 4.67 | 4.45 | 3.95 | 4.09 | 3.91 | 3.36 | BLQ | 3.08 | 3.34 | 3.73 | BLQ | 3.14 | BLQ | BLQ |
| **8** | BLQ | BLQ | BLQ | BLQ | BLQ | BLQ | BLQ | BLQ | BLQ | BLQ | BLQ | BLQ | BLQ | BLQ | BLQ | BLQ | BLQ | BLQ |
|  |  |  |  |  |  |  |  |  |  |  |  |  |  |  |  |  |  |  |
| **N** | --- | --- | 5 | 5 | 5 | 5 | 5 | 5 | 4 | 4 | --- | 4 | 4 | 5 | --- | 4 | --- | --- |
| **gMean** | --- | --- | 10.8 | 10.1 | 8.02 | 7.33 | 6.19 | 5.87 | 6.61 | 5.38 | --- | 4.38 | 4.53 | 4.54 | --- | 3.93 | --- | --- |
| **gCV [%]** | --- | --- | 78.3 | 55.4 | 51.5 | 50.4 | 55.0 | 49.3 | 40.3 | 35.4 | --- | 30.5 | 28.2 | 25.2 | --- | 20.5 | --- | --- |
| **Mean** | --- | --- | 13.3 | 11.2 | 8.77 | 7.99 | 6.87 | 6.40 | 6.97 | 5.61 | --- | 4.53 | 4.66 | 4.66 | --- | 3.99 | --- | --- |
| **CV [%]** | --- | --- | 73.3 | 46.5 | 44.9 | 44.3 | 50.2 | 46.1 | 35.1 | 31.1 | --- | 28.9 | 28.3 | 26.6 | --- | 20.4 | --- | --- |
| **SD** | --- | --- | 9.71 | 5.20 | 3.93 | 3.54 | 3.45 | 2.95 | 2.45 | 1.75 | --- | 1.31 | 1.32 | 1.24 | --- | 0.813 | --- | --- |
| **Min** | --- | --- | 5.64 | 5.62 | 4.67 | 4.45 | 3.48 | 3.40 | 3.91 | 3.36 | --- | 3.08 | 3.34 | 3.69 | --- | 3.14 | --- | --- |
| **Median** | --- | --- | 7.86 | 12.1 | 9.41 | 8.45 | 6.66 | 6.03 | 7.10 | 5.78 | --- | 4.52 | 4.43 | 4.19 | --- | 3.90 | --- | --- |
| Max | **---** | **---** | **28.4** | **17.2** | **13.8** | **12.5** | **11.9** | **10.7** | **9.77** | **7.52** | **---** | **5.99** | **6.44** | **6.66** | **---** | **5.01** | **---** | **---** |

| --- | no descriptive statistics calculated, >60% of subjects had concentrations below lower limit of quantification |
| --- | --- |
| BLQ | Below Limit of Quantification (<3.00 nmol/L) |
| BLQ excluded from descriptive statistics | |

**Supplementary Table 5:**

Individual drug plasma concentration of BI 187004 after single oral administration of 5 mg BI 187004 with descriptive statistics

| 5 mg  BI 187004 | BI 187004 plasma concentrations [nmol/L]  Planned times [h] | | | | | | | | | | | | | | | | | |
| --- | --- | --- | --- | --- | --- | --- | --- | --- | --- | --- | --- | --- | --- | --- | --- | --- | --- | --- |
| **Subject** | **–0.5** | **0.5** | **1** | **1.5** | **2** | **2.5** | **3** | **4** | **5** | **6** | **8** | **10** | **12** | **24** | **36** | **48** | **72** | **96** |
|  |  |  |  |  |  |  |  |  |  |  |  |  |  |  |  |  |  |  |
| **9** | 90.2 | BLQ | 139 | 137 | 125 | 113 | 104 | 97.2 | 88.2 | 74.1 | 67.1 | 54.2 | 51.5 | 30.9 | 14.0 | 11.6 | 5.72 | 4.37 |
| **10** | BLQ | 16.1 | 113 | 105 | 89.1 | 99.4 | 89.4 | 80.1 | 69.3 | 59.0 | 53.5 | 38.5 | 32.5 | 21.5 | 9.68 | 10.6 | 5.44 | 3.26 |
| **12** | BLQ | 180 | 184 | 143 | 140 | 139 | 130 | 124 | 109 | 90.2 | 81.4 | 58.5 | 49.0 | 33.4 | 16.9 | 10.7 | 6.47 | 4.03 |
| **13** | BLQ | 161 | 191 | 155 | 142 | 144 | 138 | 135 | 122 | 106 | 93.4 | 77.9 | 68.0 | 43.0 | 20.8 | 15.9 | 9.09 | 5.22 |
| **15** | BLQ | 128 | 142 | 124 | 109 | 120 | 107 | 95.1 | 99.2 | 77.0 | 69.8 | 55.7 | 43.3 | 21.9 | 8.76 | 8.90 | 5.40 | 4.10 |
| **16** | BLQ | 17.6 | 112 | 144 | 127 | 122 | 118 | 99.5 | 96.9 | 80.6 | 72.1 | 63.2 | 54.1 | 35.1 | 19.4 | 20.9 | 12.4 | 7.71 |
|  |  |  |  |  |  |  |  |  |  |  |  |  |  |  |  |  |  |  |
| **N** | --- | 5 | 6 | 6 | 6 | 6 | 6 | 6 | 6 | 6 | 6 | 6 | 6 | 6 | 6 | 6 | 6 | 6 |
| **gMean** | --- | 63.7 | 144 | 134 | 121 | 122 | 113 | 104 | 96.0 | 79.9 | 71.8 | 56.8 | 48.5 | 30.0 | 14.2 | 12.5 | 7.05 | 4.60 |
| **gCV [%]** | --- | 186 | 23.2 | 14.0 | 17.7 | 13.7 | 16.0 | 19.2 | 19.6 | 19.9 | 19.0 | 23.4 | 25.0 | 28.0 | 37.4 | 32.2 | 34.8 | 30.1 |
| **Mean** | --- | 101 | 147 | 135 | 122 | 123 | 114 | 105 | 97.4 | 81.2 | 72.9 | 58.0 | 49.7 | 31.0 | 14.9 | 13.1 | 7.42 | 4.78 |
| **CV [%]** | --- | 78.2 | 23.1 | 13.2 | 16.4 | 13.4 | 15.6 | 19.4 | 18.5 | 19.5 | 18.5 | 22.1 | 23.7 | 26.6 | 33.5 | 34.2 | 37.8 | 32.8 |
| **SD** | --- | 78.6 | 34.0 | 17.7 | 20.0 | 16.5 | 17.9 | 20.3 | 18.0 | 15.9 | 13.5 | 12.8 | 11.8 | 8.24 | 5.00 | 4.49 | 2.81 | 1.57 |
| **Min** | --- | 16.1 | 112 | 105 | 89.1 | 99.4 | 89.4 | 80.1 | 69.3 | 59.0 | 53.5 | 38.5 | 32.5 | 21.5 | 8.76 | 8.90 | 5.40 | 3.26 |
| **Median** | --- | 128 | 141 | 140 | 126 | 121 | 113 | 98.4 | 98.1 | 78.8 | 71.0 | 57.1 | 50.3 | 32.2 | 15.5 | 11.2 | 6.10 | 4.24 |
| Max | **---** | **180** | **191** | **155** | **142** | **144** | **138** | **135** | **122** | **106** | **93.4** | **77.9** | **68.0** | **43.0** | **20.8** | **20.9** | **12.4** | **7.71** |

| --- | no descriptive statistics calculated, >60% of subjects had concentrations below lower limit of quantification |
| --- | --- |
| BLQ | Below Limit of Quantification (<3.00 nmol/L) |
| BLQ excluded from descriptive statistics | |

**Supplementary Table 6:**

Individual drug plasma concentration of BI 187004 after single oral administration of 10 mg BI 187004 with descriptive statistics

| 10 mg  BI 187004 | BI 187004 plasma concentrations [nmol/L]  Planned times [h] | | | | | | | | | | | | | | | | | |
| --- | --- | --- | --- | --- | --- | --- | --- | --- | --- | --- | --- | --- | --- | --- | --- | --- | --- | --- |
| **Subject** | **–0.5** | **0.5** | **1** | **1.5** | **2** | **2.5** | **3** | **4** | **5** | **6** | **8** | **10** | **12** | **24** | **36** | **48** | **72** | **96** |
|  |  |  |  |  |  |  |  |  |  |  |  |  |  |  |  |  |  |  |
| **17** | BLQ | 44.7 | 397 | 437 | 403 | 362 | 323 | 280 | 223 | 214 | 170 | 118 | 115 | 46.4 | 14.1 | 12.8 | 6.84 | 3.64 |
| **18** | BLQ | 460 | 402 | 420 | 349 | 323 | 304 | 385 | 206 | 214 | 199 | 150 | 121 | 67.2 | 21.6 | 15.5 | 8.55 | 5.09 |
| **20** | BLQ | 560 | 424 | 360 | 313 | 292 | 283 | 249 | 229 | 204 | 148 | 132 | 84.7 | 39.1 | 12.8 | 11.2 | 5.11* | 3.04* |
| **22** | BLQ | 605 | 467 | 439 | 395 | 399 | 344 | 351 | 314 | 271 | 234 | 208 | 187 | 92.8 | 37.5 | 29.2 | 9.36 | 4.52 |
| **23** | BLQ | 266 | 363 | 432 | 396 | 438 | 373 | 401 | 335 | 302 | 284 | 250 | 173 | 104 | 47.4 | 32.5 | 13.9 | 7.31 |
| **24** | BLQ | 252 | 519 | 537 | 437 | 442 | 405 | 412 | 352 | 353 | 241 | 231 | 195 | 112 | 47.8 | 35.7 | 14.8 | 8.06 |
|  |  |  |  |  |  |  |  |  |  |  |  |  |  |  |  |  |  |  |
| **N** | --- | 6 | 6 | 6 | 6 | 6 | 6 | 6 | 6 | 6 | 6 | 6 | 6 | 6 | 6 | 6 | 5 | 5 |
| **gMean** | --- | 279 | 426 | 435 | 380 | 372 | 336 | 340 | 270 | 254 | 208 | 174 | 140 | 71.3 | 26.3 | 20.5 | 10.2 | 5.48 |
| **gCV [%]** | --- | 125 | 12.8 | 12.8 | 12.0 | 16.9 | 13.3 | 21.0 | 23.8 | 22.6 | 24.5 | 32.2 | 34.1 | 46.1 | 65.3 | 54.7 | 33.8 | 34.2 |
| **Mean** | --- | 365 | 429 | 438 | 382 | 376 | 339 | 346 | 277 | 260 | 213 | 182 | 146 | 76.9 | 30.2 | 22.8 | 10.7 | 5.72 |
| **CV [%]** | --- | 58.8 | 13.1 | 13.0 | 11.5 | 16.3 | 13.3 | 19.5 | 23.2 | 23.0 | 23.5 | 30.5 | 30.9 | 39.7 | 53.3 | 47.6 | 32.5 | 32.9 |
| **SD** | --- | 214 | 56.0 | 57.0 | 44.0 | 61.3 | 45.1 | 67.4 | 64.2 | 59.8 | 50.1 | 55.4 | 45.1 | 30.6 | 16.1 | 10.9 | 3.48 | 1.88 |
| **Min** | --- | 44.7 | 363 | 360 | 313 | 292 | 283 | 249 | 206 | 204 | 148 | 118 | 84.7 | 39.1 | 12.8 | 11.2 | 6.84 | 3.64 |
| **Median** | --- | 363 | 413 | 435 | 396 | 381 | 334 | 368 | 272 | 243 | 217 | 179 | 147 | 80.0 | 29.6 | 22.4 | 9.36 | 5.09 |
| Max | **---** | **605** | **519** | **537** | **437** | **442** | **405** | **412** | **352** | **353** | **284** | **250** | **195** | **112** | **47.8** | **35.7** | **14.8** | **8.06** |

| --- | no descriptive statistics calculated, >60% of subjects had concentrations below lower limit of quantification |
| --- | --- |
| * | excluded from descriptive statistics |
| BLQ | Below Limit of Quantification (<3.00 nmol/L) |
| BLQ excluded from descriptive statistics | |

**Supplementary Table 7:**

Individual drug plasma concentration of BI 187004 after single oral administration of 20 mg BI 187004 with descriptive statistics

| 20 mg  BI 187004 | BI 187004 plasma concentrations [nmol/L]  Planned times [h] | | | | | | | | | | | | | | | | | |
| --- | --- | --- | --- | --- | --- | --- | --- | --- | --- | --- | --- | --- | --- | --- | --- | --- | --- | --- |
| **Subject** | **–0.5** | **0.5** | **1** | **1.5** | **2** | **2.5** | **3** | **4** | **5** | **6** | **8** | **10** | **12** | **24** | **36** | **48** | **72** | **96** |
|  |  |  |  |  |  |  |  |  |  |  |  |  |  |  |  |  |  |  |
| **25** | BLQ | 34.3 | 236 | 919 | 832 | 783 | 663 | 663 | 512 | 447 | 378 | 335 | 251 | 107 | 33.6 | 21.7 | 9.53 | 6.00 |
| **26** | BLQ | 80.7 | 1100 | 842 | 784 | 710 | 586 | 537 | 463 | 420 | 359 | 253 | 234 | 95.1 | 27.8 | 19.4 | BLQ | 5.78 |
| **28** | BLQ | 338 | 1130 | 1050 | 966 | 1040 | 877 | 885 | 883 | 611 | 631 | 529 | 457 | 281 | 96.8 | 65.7 | 25.9 | 12.4 |
| **30** | BLQ | 91.1 | 750 | 824 | 630 | 619 | 610 | 531 | 535 | 427 | 418 | 288 | 221 | 85.8 | 32.3 | 15.8 | 6.98 | BLQ |
| **31** | BLQ | 1050 | 965 | 969 | 947 | 948 | 879 | 833 | 753 | 646 | 354 | 461 | 370 | 203 | 68.5 | 47.0 | 16.4 | 9.48 |
| **32** | BLQ | 989 | 1130 | 1210 | 1100 | 1060 | 945 | 896 | 773 | 847 | 645 | 524 | 450 | 353 | 145 | 104 | 43.9 | 18.2 |
|  |  |  |  |  |  |  |  |  |  |  |  |  |  |  |  |  |  |  |
| **N** | --- | 6 | 6 | 6 | 6 | 6 | 6 | 6 | 6 | 6 | 6 | 6 | 6 | 6 | 6 | 6 | 5 | 5 |
| **gMean** | --- | 211 | 788 | 960 | 863 | 843 | 746 | 707 | 634 | 547 | 449 | 382 | 316 | 161 | 55.4 | 35.9 | 16.5 | 9.42 |
| **gCV [%]** | --- | 256 | 67.3 | 14.5 | 19.7 | 22.2 | 21.3 | 24.7 | 27.0 | 29.0 | 28.4 | 32.7 | 34.3 | 66.3 | 76.4 | 88.2 | 85.7 | 51.8 |
| **Mean** | --- | 431 | 885 | 969 | 877 | 860 | 760 | 724 | 653 | 566 | 464 | 398 | 331 | 187 | 67.3 | 45.6 | 20.5 | 10.4 |
| **CV [%]** | --- | 109 | 39.5 | 14.9 | 18.7 | 21.2 | 20.7 | 23.4 | 26.3 | 29.8 | 29.4 | 30.6 | 33.0 | 59.3 | 69.1 | 75.7 | 72.9 | 49.7 |
| **SD** | --- | 469 | 350 | 144 | 164 | 183 | 158 | 169 | 172 | 169 | 137 | 122 | 109 | 111 | 46.5 | 34.5 | 15.0 | 5.16 |
| **Min** | --- | 34.3 | 236 | 824 | 630 | 619 | 586 | 531 | 463 | 420 | 354 | 253 | 221 | 85.8 | 27.8 | 15.8 | 6.98 | 5.78 |
| **Median** | --- | 215 | 1030 | 944 | 890 | 866 | 770 | 748 | 644 | 529 | 398 | 398 | 311 | 155 | 51.1 | 34.4 | 16.4 | 9.48 |
| Max | **---** | **1050** | **1130** | **1210** | **1100** | **1060** | **945** | **896** | **883** | **847** | **645** | **529** | **457** | **353** | **145** | **104** | **43.9** | **18.2** |

| --- | no descriptive statistics calculated, >60% of subjects had concentrations below lower limit of quantification |
| --- | --- |
| BLQ | Below Limit of Quantification (<3.00 nmol/L) |
| BLQ excluded from descriptive statistics | |

**Supplementary Table 8:**

Individual drug plasma concentration of BI 187004 after single oral administration of 40 mg BI 187004 with descriptive statistics

| 40 mg  BI 187004 | BI 187004 plasma concentrations [nmol/L]  Planned times [h] | | | | | | | | | | | | | | | | | |
| --- | --- | --- | --- | --- | --- | --- | --- | --- | --- | --- | --- | --- | --- | --- | --- | --- | --- | --- |
| **Subject** | **–0.5** | **0.5** | **1** | **1.5** | **2** | **2.5** | **3** | **4** | **5** | **6** | **8** | **10** | **12** | **24** | **36** | **48** | **72** | **96** |
|  |  |  |  |  |  |  |  |  |  |  |  |  |  |  |  |  |  |  |
| **33** | BLQ | 2400 | 2490 | 2240 | 1900 | 2010 | 1680 | 1650 | 1610 | 1320 | 1190 | 1140 | 984 | 625 | 377 | 234 | 83.4 | 41.4 |
| **34** | BLQ | 2460 | 2320 | 2220 | 2020 | 1920 | 1810 | 1700 | 1550 | 1400 | 1100 | 975 | 838 | 436 | 244 | 143 | 41.8 | 16.0 |
| **36** | BLQ | 1110 | 2690 | 2090 | 1820 | 1820 | 1440 | 1410 | 1160 | 985 | 834 | 738 | 538 | 215 | 70.1 | 30.8 | 8.48 | 5.05 |
| **38** | BLQ | 487 | 974 | 1020 | 1040 | 1160 | 1430 | 1320 | 1250 | 1020 | 973 | 823 | 716 | 379 | 138 | 72.0 | 22.0 | 8.34 |
| **39** | BLQ | 1560 | 1730 | 1540 | 1360 | 1420 | 1130 | 1020 | 888 | 736 | 606 | 467 | 359 | 169 | 51.5 | 33.7 | 14.2 | 8.88 |
| **40** | BLQ | 2530 | 2820 | 2610 | 2410 | 2180 | 1680 | 1640 | 1440 | 1230 | 1000 | 806 | 663 | 291 | 99.1 | 60.3 | 22.1 | 13.3 |
|  |  |  |  |  |  |  |  |  |  |  |  |  |  |  |  |  |  |  |
| **N** | --- | 6 | 6 | 6 | 6 | 6 | 6 | 6 | 6 | 6 | 6 | 6 | 6 | 6 | 6 | 6 | 6 | 6 |
| **gMean** | --- | 1520 | 2050 | 1870 | 1700 | 1710 | 1510 | 1440 | 1290 | 1090 | 929 | 796 | 650 | 321 | 129 | 73.0 | 24.3 | 12.2 |
| **gCV [%]** | --- | 72.3 | 42.0 | 35.4 | 31.0 | 24.3 | 17.1 | 19.7 | 22.5 | 24.1 | 24.5 | 31.1 | 36.8 | 50.8 | 87.6 | 94.4 | 95.3 | 82.5 |
| **Mean** | --- | 1760 | 2170 | 1950 | 1760 | 1750 | 1530 | 1460 | 1320 | 1120 | 951 | 825 | 683 | 353 | 163 | 95.6 | 32.0 | 15.5 |
| **CV [%]** | --- | 48.2 | 32.2 | 29.4 | 27.8 | 22.0 | 16.1 | 17.9 | 20.6 | 22.2 | 21.8 | 27.5 | 32.2 | 47.2 | 76.6 | 82.6 | 86.2 | 85.6 |
| **SD** | --- | 847 | 699 | 574 | 488 | 386 | 246 | 261 | 271 | 247 | 207 | 227 | 220 | 166 | 125 | 79.0 | 27.6 | 13.3 |
| **Min** | --- | 487 | 974 | 1020 | 1040 | 1160 | 1130 | 1020 | 888 | 736 | 606 | 467 | 359 | 169 | 51.5 | 30.8 | 8.48 | 5.05 |
| **Median** | --- | 1980 | 2410 | 2160 | 1860 | 1870 | 1560 | 1530 | 1350 | 1130 | 987 | 815 | 690 | 335 | 119 | 66.2 | 22.1 | 11.1 |
| Max | **---** | **2530** | **2820** | **2610** | **2410** | **2180** | **1810** | **1700** | **1610** | **1400** | **1190** | **1140** | **984** | **625** | **377** | **234** | **83.4** | **41.4** |

| --- | no descriptive statistics calculated, >60% of subjects had concentrations below lower limit of quantification |
| --- | --- |
| BLQ | Below Limit of Quantification (<3.00 nmol/L) |
| BLQ excluded from descriptive statistics | |

**Supplementary Table 9:**

Individual drug plasma concentration of BI 187004 after single oral administration of 80 mg BI 187004 with descriptive statistics

| 80 mg  BI 187004 | BI 187004 plasma concentrations [nmol/L]  Planned times [h] | | | | | | | | | | | | | | | | | |
| --- | --- | --- | --- | --- | --- | --- | --- | --- | --- | --- | --- | --- | --- | --- | --- | --- | --- | --- |
| **Subject** | **–0.5** | **0.5** | **1** | **1.5** | **2** | **2.5** | **3** | **4** | **5** | **6** | **8** | **10** | **12** | **24** | **36** | **48** | **72** | **96** |
|  |  |  |  |  |  |  |  |  |  |  |  |  |  |  |  |  |  |  |
| **41** | BLQ | 1310 | 1380 | 1380 | 1460 | 1450 | 1360 | 1380 | 1350 | 1210 | 1420 | 1200 | 959 | 605 | 284 | 163 | 49.0 | 18.8 |
| **43** | BLQ | 738 | 2340 | 2790 | 2570 | 2940 | 2570 | 2750 | 2080 | 2080 | 2190 | 1870 | 1680 | 1100 | 538 | 332 | 81.2 | 24.3 |
| **44** | BLQ | 4480 | 4770 | 4150 | 3590 | 3200 | 3340 | 3200 | 3210 | 2460 | 2840 | 2570 | 2210 | 1560 | 678 | 596 | 234 | 110 |
| **45** | BLQ | 539 | 2250 | 2290 | 2230 | 2640 | 2360 | 2220 | 2000 | 1750 | 1630 | 1370 | 1190 | 856 | 417 | 248 | 82.3 | 81.0 |
| **46** | BLQ | 3280 | 3430 | 3410 | 3110 | 2650 | 2750 | 2700 | 2350 | 1990 | 1750 | 1540 | 1230 | 673 | 327 | 211 | 46.9 | 11.9 |
| **47** | BLQ | 304 | 771 | 958 | 1000 | 1200 | 1050 | 936 | 905 | 930 | 1100 | 1040 | 1000 | 767 | 223 | 134 | 46.3 | 23.3 |
|  |  |  |  |  |  |  |  |  |  |  |  |  |  |  |  |  |  |  |
| **N** | --- | 6 | 6 | 6 | 6 | 6 | 6 | 6 | 6 | 6 | 6 | 6 | 6 | 6 | 6 | 6 | 6 | 6 |
| **gMean** | --- | 1150 | 2120 | 2220 | 2130 | 2200 | 2070 | 2020 | 1840 | 1650 | 1740 | 1530 | 1320 | 878 | 383 | 247 | 74.2 | 32.3 |
| **gCV [%]** | --- | 142 | 72.5 | 60.5 | 51.3 | 42.6 | 47.3 | 50.5 | 46.8 | 38.1 | 34.1 | 33.4 | 33.1 | 36.0 | 43.2 | 57.7 | 68.9 | 107 |
| **Mean** | --- | 1780 | 2490 | 2500 | 2330 | 2350 | 2240 | 2200 | 1980 | 1740 | 1820 | 1600 | 1380 | 927 | 411 | 281 | 90.0 | 44.9 |
| **CV [%]** | --- | 96.1 | 57.8 | 48.4 | 42.1 | 35.0 | 38.9 | 39.8 | 40.4 | 32.9 | 33.8 | 34.8 | 34.9 | 38.3 | 41.6 | 60.3 | 80.7 | 90.2 |
| **SD** | --- | 1710 | 1440 | 1210 | 979 | 822 | 870 | 874 | 802 | 572 | 616 | 556 | 481 | 355 | 171 | 169 | 72.6 | 40.5 |
| **Min** | --- | 304 | 771 | 958 | 1000 | 1200 | 1050 | 936 | 905 | 930 | 1100 | 1040 | 959 | 605 | 223 | 134 | 46.3 | 11.9 |
| **Median** | --- | 1020 | 2300 | 2540 | 2400 | 2650 | 2470 | 2460 | 2040 | 1870 | 1690 | 1460 | 1210 | 812 | 372 | 230 | 65.1 | 23.8 |
| Max | **---** | **4480** | **4770** | **4150** | **3590** | **3200** | **3340** | **3200** | **3210** | **2460** | **2840** | **2570** | **2210** | **1560** | **678** | **596** | **234** | **110** |

| --- | no descriptive statistics calculated, >60% of subjects had concentrations below lower limit of quantification |
| --- | --- |
| BLQ | Below Limit of Quantification (<3.00 nmol/L) |
| BLQ excluded from descriptive statistics | |

**Supplementary Table 10:**

Individual drug plasma concentration of BI 187004 after single oral administration of 160 mg BI 187004 with descriptive statistics

| 160 mg  BI 187004 | BI 187004 plasma concentrations [nmol/L]  Planned times [h] | | | | | | | | | | | | | | | | | |
| --- | --- | --- | --- | --- | --- | --- | --- | --- | --- | --- | --- | --- | --- | --- | --- | --- | --- | --- |
| **Subject** | **–0.5** | **0.5** | **1** | **1.5** | **2** | **2.5** | **3** | **4** | **5** | **6** | **8** | **10** | **12** | **24** | **36** | **48** | **72** | **96** |
|  |  |  |  |  |  |  |  |  |  |  |  |  |  |  |  |  |  |  |
| **49** | BLQ | 8780 | 7170 | 7110 | 6610 | 6250 | 5820 | 6210 | 5220 | 3980 | 2580 | 3120 | 2810 | 1530 | 655 | 333 | 113 | 28.0 |
| **50** | BLQ | 4660 | 7510 | 8830 | 8640 | 8030 | 7450 | 6040 | 4630 | 4420 | 4030 | 3410 | 2940 | 1410 | 576 | 359 | 103 | 32.1 |
| **52** | BLQ | 8640 | 7770 | 7270 | 6360 | 6070 | 5770 | 5530 | 5160 | 4830 | 3800 | 2810 | 2920 | 1660 | 476 | 374 | 121 | 46.8 |
| **54** | BLQ | 7950 | 8740 | 7780 | 6910 | 6820 | 5860 | 6750 | 4950 | 5320 | 4580 | 4290 | 3640 | 2690 | 1390 | 1000 | 312 | BLQ |
| **55** | BLQ | 2520 | 3920 | 5190 | 6660 | 6270 | 5330 | 5060 | 4850 | 3430 | 3680 | 1940 | 2890 | 1440 | 631 | 329 | 85.9 | 31.7 |
| **56** | BLQ | 5710 | 8460 | 7480 | 7230 | 6930 | 5470 | 5230 | 4420 | 4030 | 3650 | 2950 | 2710 | 1360 | 570 | 344 | 100 | 30.2 |
|  |  |  |  |  |  |  |  |  |  |  |  |  |  |  |  |  |  |  |
| **N** | --- | 6 | 6 | 6 | 6 | 6 | 6 | 6 | 6 | 6 | 6 | 6 | 6 | 6 | 6 | 6 | 6 | 5 |
| **gMean** | --- | 5860 | 7040 | 7190 | 7030 | 6700 | 5910 | 5770 | 4860 | 4290 | 3670 | 3000 | 2970 | 1630 | 669 | 414 | 125 | 33.2 |
| **gCV [%]** | --- | 51.4 | 30.2 | 17.8 | 11.0 | 10.3 | 12.0 | 11.1 | 6.39 | 15.6 | 19.3 | 26.5 | 10.4 | 25.9 | 38.8 | 45.6 | 49.0 | 20.1 |
| **Mean** | --- | 6380 | 7260 | 7280 | 7070 | 6730 | 5950 | 5800 | 4870 | 4340 | 3720 | 3090 | 2990 | 1680 | 716 | 457 | 139 | 33.8 |
| **CV [%]** | --- | 39.4 | 23.9 | 16.4 | 11.7 | 10.7 | 12.8 | 11.1 | 6.32 | 15.5 | 17.6 | 24.9 | 11.1 | 30.0 | 46.9 | 58.4 | 61.5 | 22.1 |
| **SD** | --- | 2520 | 1740 | 1190 | 825 | 723 | 764 | 644 | 308 | 672 | 655 | 770 | 332 | 505 | 336 | 267 | 85.5 | 7.46 |
| **Min** | --- | 2520 | 3920 | 5190 | 6360 | 6070 | 5330 | 5060 | 4420 | 3430 | 2580 | 1940 | 2710 | 1360 | 476 | 329 | 85.9 | 28.0 |
| **Median** | --- | 6830 | 7640 | 7380 | 6790 | 6550 | 5800 | 5790 | 4900 | 4230 | 3740 | 3040 | 2910 | 1490 | 604 | 352 | 108 | 31.7 |
| Max | **---** | **8780** | **8740** | **8830** | **8640** | **8030** | **7450** | **6750** | **5220** | **5320** | **4580** | **4290** | **3640** | **2690** | **1390** | **1000** | **312** | **46.8** |

| --- | no descriptive statistics calculated, >60% of subjects had concentrations below lower limit of quantification |
| --- | --- |
| BLQ | Below Limit of Quantification (<3.00 nmol/L) |
| BLQ excluded from descriptive statistics | |

**Supplementary Table 11:**

Individual drug plasma concentration of BI 187004 after single oral administration of 240 mg BI 187004 with descriptive statistics

| 240 mg  BI 187004 | BI 187004 plasma concentrations [nmol/L]  Planned times [h] | | | | | | | | | | | | | | | | | |
| --- | --- | --- | --- | --- | --- | --- | --- | --- | --- | --- | --- | --- | --- | --- | --- | --- | --- | --- |
| **Subject** | **-0.5** | **0.5** | **1** | **1.5** | **2** | **2.5** | **3** | **4** | **5** | **6** | **8** | **10** | **12** | **24** | **36** | **48** | **72** | **96** |
|  |  |  |  |  |  |  |  |  |  |  |  |  |  |  |  |  |  |  |
| **58** | BLQ | 7450 | 10700 | 8600 | 9220 | 8490 | 6980 | 5540 | 3960 | 5150 | 4780 | 4080 | 3440 | 2500 | 1200 | 755 | 323 | 181 |
| **59** | BLQ | 4950 | 9740 | 9030 | 8860 | 8510 | 5360 | 8420 | 6570 | 6370 | 5380 | 5800 | 3900 | 2630 | 1340 | 794 | 312 | 68.2 |
| **60** | BLQ | 6100 | 7600 | 7090 | 6350 | 6340 | 6510 | 5820 | 5820 | 5390 | 3960 | 3310 | 3170 | 1720 | 787 | 376 | 54.4 | 18.4* |
| **61** | BLQ | 8440 | 9250 | 6280 | 5400 | 5680 | 5530 | 5390 | 5040 | 2610 | 3640 | 3060 | 2610 | 1530 | 677 | 321 | 71.7 | 18.2 |
| **62** | BLQ | 4180 | 7180 | 6690 | 7210 | 8190 | 6150 | 7330 | 6780 | 5990 | 5130 | 4160 | 2180 | 2460 | 987 | 545 | 126 | 36.4 |
| **64** | BLQ | 130 | 440 | 811 | 543 | 605 | 388 | 575 | 494 | 452 | 181 | 313 | 258 | 2180 | 567 | 255 | 54.1 | 15.8 |
|  |  |  |  |  |  |  |  |  |  |  |  |  |  |  |  |  |  |  |
| **N** | --- | 6 | 6 | 6 | 6 | 6 | 6 | 6 | 6 | 6 | 6 | 6 | 6 | 6 | 6 | 6 | 6 | 5 |
| **gMean** | --- | 3180 | 5340 | 5150 | 4710 | 4840 | 3840 | 4280 | 3700 | 3280 | 2650 | 2600 | 1990 | 2130 | 885 | 465 | 118 | 41.9 |
| **gCV [%]** | --- | 338 | 189 | 115 | 148 | 138 | 160 | 131 | 132 | 136 | 218 | 144 | 136 | 22.5 | 34.4 | 49.3 | 99.1 | 132 |
| **Mean** | --- | 5210 | 7490 | 6420 | 6260 | 6300 | 5150 | 5510 | 4780 | 4330 | 3850 | 3450 | 2590 | 2170 | 926 | 508 | 157 | 63.9 |
| **CV [%]** | --- | 56.4 | 49.4 | 46.0 | 50.4 | 48.2 | 46.8 | 48.8 | 49.0 | 53.4 | 49.8 | 52.5 | 49.9 | 20.8 | 32.8 | 45.0 | 81.1 | 108 |
| **SD** | --- | 2940 | 3700 | 2950 | 3160 | 3040 | 2410 | 2690 | 2340 | 2310 | 1920 | 1810 | 1290 | 451 | 303 | 228 | 127 | 68.7 |
| **Min** | --- | 130 | 440 | 811 | 543 | 605 | 388 | 575 | 494 | 452 | 181 | 313 | 258 | 1530 | 567 | 255 | 54.1 | 15.8 |
| **Median** | --- | 5530 | 8430 | 6890 | 6780 | 7270 | 5840 | 5680 | 5430 | 5270 | 4370 | 3700 | 2890 | 2320 | 887 | 461 | 98.9 | 36.4 |
| Max | **---** | **8440** | **10700** | **9030** | **9220** | **8510** | **6980** | **8420** | **6780** | **6370** | **5380** | **5800** | **3900** | **2630** | **1340** | **794** | **323** | **181** |

| --- | no descriptive statistics calculated, >60% of subjects had concentrations below lower limit of quantification |
| --- | --- |
| BLQ | Below Limit of Quantification (<3.00 nmol/L) |
| BLQ excluded from descriptive statistics | |

**Supplementary Table 12:**

Individual drug plasma concentration of BI 187004 after single oral administration of 360 mg BI 187004 with descriptive statistics

| 360 mg  BI 187004 | BI 187004 plasma concentrations [nmol/L]  Planned times [h] | | | | | | | | | | | | | | | | | |
| --- | --- | --- | --- | --- | --- | --- | --- | --- | --- | --- | --- | --- | --- | --- | --- | --- | --- | --- |
| **Subject** | **–0.5** | **0.5** | **1** | **1.5** | **2** | **2.5** | **3** | **4** | **5** | **6** | **8** | **10** | **12** | **24** | **36** | **48** | **72** | **96** |
|  |  |  |  |  |  |  |  |  |  |  |  |  |  |  |  |  |  |  |
| **65** | BLQ | 16800 | 12000 | 10800 | 10700 | 9770 | 9220 | 8290 | 8770 | 8320 | 7780 | 6390 | 5510 | 3110 | 1990 | 1220 | 516 | 170 |
| **67** | BLQ | 11000 | 14500 | 13900 | 10900 | 14100 | 7970 | 10400 | 8180 | 6450 | 6280 | 4960 | 4380 | 1790 | 830 | 357 | 69.1 | 16.8 |
| **68** | BLQ | 5800 | 7170 | 6450 | 6660 | 6880 | 6300 | 5670 | 4810 | 5790 | 5060 | 3360 | 3780 | 2300 | 1300 | 740 | 235 | 61.1 |
| **70** | BLQ | 7980 | 13600 | 12400 | 10400 | 5660 | 9450 | 10100 | 7560 | 6450 | 6260 | 6280 | 4600 | 3010 | 1680 | 997 | 326 | 102 |
| **71** | BLQ | 12200 | 13000 | 9540 | 12300 | 10700 | 10300 | 10900 | 9630 | 6310 | 6210 | 4310 | 4650 | 2090 | 1090 | 587 | 164 | 46.3 |
| **72** | BLQ | 15400 | 18400 | 15900 | 14500 | 14600 | 12900 | 12200 | 6320 | 10200 | 9510 | 7680 | 6080 | 4740 | 1970 | 1470 | 497 | 174 |
|  |  |  |  |  |  |  |  |  |  |  |  |  |  |  |  |  |  |  |
| **N** | --- | 6 | 6 | 6 | 6 | 6 | 6 | 6 | 6 | 6 | 6 | 6 | 6 | 6 | 6 | 6 | 6 | 6 |
| **gMean** | --- | 10800 | 12600 | 11100 | 10600 | 9710 | 9140 | 9320 | 7360 | 7110 | 6710 | 5300 | 4780 | 2690 | 1410 | 814 | 246 | 72.3 |
| **gCV [%]** | --- | 42.1 | 32.1 | 32.8 | 26.4 | 39.5 | 24.5 | 28.0 | 25.7 | 21.7 | 22.1 | 30.9 | 17.0 | 36.0 | 36.2 | 53.8 | 88.7 | 110 |
| **Mean** | --- | 11500 | 13100 | 11500 | 10900 | 10300 | 9360 | 9590 | 7550 | 7250 | 6850 | 5500 | 4830 | 2840 | 1480 | 898 | 301 | 95.0 |
| **CV [%]** | --- | 36.6 | 27.9 | 29.0 | 23.6 | 35.5 | 23.8 | 24.0 | 23.1 | 23.2 | 22.8 | 28.7 | 17.1 | 37.5 | 32.4 | 45.5 | 59.8 | 69.1 |
| **SD** | --- | 4220 | 3650 | 3340 | 2580 | 3650 | 2220 | 2300 | 1750 | 1680 | 1560 | 1580 | 826 | 1060 | 479 | 409 | 180 | 65.7 |
| **Min** | --- | 5800 | 7170 | 6450 | 6660 | 5660 | 6300 | 5670 | 4810 | 5790 | 5060 | 3360 | 3780 | 1790 | 830 | 375 | 69.1 | 16.8 |
| **Median** | --- | 11600 | 13300 | 11600 | 10800 | 10200 | 9340 | 10300 | 7870 | 6450 | 6270 | 5620 | 4630 | 2660 | 1490 | 869 | 281 | 81.6 |
| Max | **---** | **16800** | **18400** | **15900** | **14500** | **14600** | **12900** | **12200** | **9630** | **10200** | **9510** | **7680** | **6080** | **4740** | **1990** | **1470** | **516** | **174** |

| --- | no descriptive statistics calculated, >60% of subjects had concentrations below lower limit of quantification |
| --- | --- |
| BLQ | Below Limit of Quantification (<3.00 nmol/L) |
| BLQ excluded from descriptive statistics | |

**Supplementary Table 13:**

Individual THF/THE ratio at baseline and after single ascending dose administrations in healthy volunteers

| BI 187004 |  | (aTHF + THF)/THE | |
| --- | --- | --- | --- |
| **Dose group** | **Subject** | **Baseline** | **24 h** |
| **2.5 mg** | 2 | 1.28 | 0.692 |
|  | 3 | 1.36 | 0.456 |
|  | 4 | 1.40 | 0.648 |
|  | 5 | 1.12 | 0.500 |
|  | 7 | 1.10 | 0.585 |
|  | 8 | 1.12 | 0.665 |
| **5 mg** | 9 | 1.26 | 0.407 |
|  | 10 | 1.65 | 0.379 |
|  | 12 | 1.66 | 0.340 |
|  | 13 | 1.65 | 0.479 |
|  | 15 | 1.63 | 0.523 |
|  | 16 | 1.12 | 0.455 |
| **10 mg** | 17 | 1.41 | 0.509 |
|  | 18 | 1.46 | 0.382 |
|  | 20 | 1.41 | 0.451 |
|  | 22 | 1.80 | 0.382 |
|  | 23 | 1.36 | 0.432 |
|  | 24 | 1.20 | 0.440 |
| **20 mg** | 25 | 1.23 | 0.409 |
|  | 26 | 1.93 | 0.461 |
|  | 28 | 1.26 | 0.349 |
|  | 30 | 1.32 | 0.383 |
|  | 31 | 1.50 | 0.423 |
|  | 32 | 1.78 | 0.518 |
| **40 mg** | 33 | 1.02 | 0.323 |
|  | 34 | 1.40 | 0.371 |
|  | 36 | 0.862 | 0.304 |
|  | 38 | 1.28 | 0.452 |
|  | 39 | 1.28 | 0.363 |
|  | 40 | 1.26 | 0.409 |
| **80 mg** | 41 | 1.22 | 0.372 |
|  | 43 | 1.42 | 0.495 |
|  | 44 | 0.893 | 0.337 |
|  | 45 | 1.93 | 0.515 |
|  | 46 | 1.17 | 0.355 |
|  | 47 | 1.20 | 0.418 |
| **160 mg** | 49 | 1.22 | 0.485 |
|  | 50 | 1.43 | 0.436 |
|  | 52 | 1.25 | 0.306 |
|  | 54 | 1.37 | 0.450 |
|  | 55 | 1.18 | 0.392 |
|  | 56 | 1.21 | 0.474 |
| **240 mg** | 58 | 1.65 | 0.455 |
|  | 59 | 1.35 | 0.463 |
|  | 60 | 1.23 | 0.433 |
|  | 61 | 1.53 | 0.485 |
|  | 62 | 1.37 | 0.483 |
|  | 64 | 2.05 | 0.538 |

| BI 187004 |  | (aTHF + THF)/THE | |
| --- | --- | --- | --- |
| **Dose group** | **Subject** | **Baseline** | **24 h** |
| **360 mg** | 65 | 1.43 | 0.571 |
|  | 67 | 1.14 | 0.356 |
|  | 68 | 1.17 | 0.377 |
|  | 70 | 1.68 | 0.451 |
|  | 71 | 1.43 | 0.478 |
|  | 72 | 1.22 | 0.352 |
| **Placebo** | 1 | 1.38 | 1.34 |
|  | 6 | 1.71 | 1.54 |
|  | 11 | 1.16 | 1.22 |
|  | 19 | 1.45 | 1.25 |
|  | 21 | 1.26 | 1.34 |
|  | 27 | 1.48 | 1.56 |
|  | 29 | 1.36 | 1.34 |
|  | 35 | 1.11 | 1.06 |
|  | 37 | 1.36 | 1.58 |
|  | 42 | 1.30 | 1.21 |
|  | 48 | 1.35 | 1.48 |
|  | 51 | 1.25 | 1.23 |
|  | 53 | 1.50 | 1.33 |
|  | 57 | 1.75 | 1.74 |
|  | 63 | 1.79 | 1.75 |
|  | 66 | 1.34 | 1.34 |
|  | 69 | 1.10 | 1.15 |

**Supplementary Table 14:**

Individual cortisol levels in adipose tissue at baseline, 10 h and 24 h after single ascending dose administrations in healthy volunteers.

| BI 187004 |  | Cortisol [ng/ml/g] | | |
| --- | --- | --- | --- | --- |
| **Dose group** | **Subject** | **Baseline** | **10 h** | **24 h** |
| **10 mg** | 17 | 258 | 22.3 | 113 |
|  | 18 | 80.5 | 15.2 | 53.1 |
|  | 20 | 202 | 26.0 | 77.3 |
|  | 22 | 236 | 43.4 | 93.0 |
|  | 23 | 166 | 22.6 | 35.6 |
|  | 24 | 373 | 44.7 | 156 |
| **20 mg** | 25 | 699 | 127.0 | 114 |
|  | 26 | 205 | 63.5 | 55.0 |
|  | 28 | 424 | 79.6 | 81.3 |
|  | 30 | 408 | 45.1 | 143.0 |
|  | 31 | 191 | 60.2 | 27.2 |
|  | 32 | 302 | 19.2 | 28.0 |
| **40 mg** | 33 | 661 | 23.1 | 37.5 |
|  | 34 | 294 | 9.81 | 14.7 |
|  | 36 | 152 | 9.66 | 24.2 |
|  | 38 | 320 | 15.2 | 54.6 |
|  | 39 | 229 | 9.26 | 38.9 |
|  | 40 | 228 | 11.4 | 33.8 |
| **80 mg** | 41 | 377 | 10.6 | 24.5 |
|  | 43 | 387 | 13.9 | 12.8 |
|  | 44 | 472 | 5.90 | 14.6 |
|  | 45 | 393 | 16.9 | 25.4 |
|  | 46 | 641 | 7.62 | 57.0 |
|  | 47 | 101 | 2.27 | 8.08 |
| **160 mg** | 49 | 453 | 4.04 | 7.93 |
|  | 50 | 231 | 1.87 | 13.8 |
|  | 52 | 292 | 2.58 | 6.25 |
|  | 54 | 236 | 1.53 | 2.60 |
|  | 55 | 315 | 3.04 | 6.23 |
|  | 56 | 291 | 3.49 | 10.8 |
| **240 mg** | 58 | 281 | 1.33 | 3.51 |
|  | 59 | 368 | 1.54 | 6.86 |
|  | 60 | 214 | 1.84 | 6.86 |
|  | 61 | 252 | 2.25 | 8.18 |
|  | 62 | 489 | 3.09 | 8.41 |
|  | 64 | 162 | 0.927 | 3.00 |
| **360 mg** | 65 | 339 | 1.67 | 6.35 |
|  | 67 | 458 | 3.14 | 8.97 |
|  | 68 | 411 | 2.09 | 5.42 |
|  | 70 | 351 | 1.56 | 5.47 |
|  | 71 | 507 | 1.82 | 3.20 |
|  | 72 | 153 | 0.684 | 1.70 |
| **Placebo** | 19 | 269 | 338 | 273 |
|  | 21 | 339 | 215 | 193 |
|  | 27 | 367 | 394 | 211 |
|  | 29 | 244 | 172 | 236 |
|  | 35 | 389 | 576 | 650 |
|  | 37 | 483 | 411 | 331 |
|  | 42 | 209 | 168 | 210 |
|  | 48 | 436 | 416 | 503 |
|  | 51 | 398 | 466 | 317 |
|  | 53 | 359 | 473 | 424 |
|  | 57 | 260 | 311 | 203 |
|  | 63 | 304 | 312 | 358 |
|  | 66 | 240 | 218 | 193 |
|  | 69 | 300 | 349 | 303 |
